# Structure, Mechanics, and Mechanobiology of Fibrocartilage Pericellular Matrix Mediated by Type V Collagen

**DOI:** 10.1002/advs.202414750

**Published:** 2025-05-23

**Authors:** Chao Wang, Mingyue Fan, Su Chin Heo, Sheila M. Adams, Thomas Li, Yuchen Liu, Qing Li, Claudia Loebel, Jason A. Burdick, X. Lucas Lu, David E. Birk, Farid Alisafaei, Robert L. Mauck, Lin Han

**Affiliations:** ^1^ School of Biomedical Engineering Science and Health Systems Drexel University Philadelphia PA 19104 USA; ^2^ McKay Orthopaedic Research Laboratory Department of Orthopaedic Surgery Perelman School of Medicine University of Pennsylvania Philadelphia PA 19104 USA; ^3^ Department of Molecular Pharmacology and Physiology Morsani School of Medicine University of South Florida Tampa FL 33612 USA; ^4^ Department of Bioengineering University of Pennsylvania Philadelphia PA 19104 USA; ^5^ BioFrontiers Institute and Department of Chemical and Biological Engineering University of Colorado Boulder CO 80309 USA; ^6^ Department of Mechanical Engineering University of Delaware Newark DE 19716 USA; ^7^ Department of Mechanical and Industrial Engineering New Jersey Institute of Technology Newark NJ 07102 USA; ^8^ Translational Musculoskeletal Research Center Corporal Michael J. Crescenz Veterans Administration Medical Center Philadelphia PA 19104 USA

**Keywords:** fibrocartilage, mechanotransduction, micromechanics, pericellular matrix, type V collagen

## Abstract

The pericellular matrix (PCM) is the immediate microniche surrounding cells in various tissues, regulating matrix turnover, cell‐matrix interactions, and disease. This study elucidates the structure‐mechanical properties and mechanobiology of the PCM in fibrocartilage, using the murine meniscus as the model. The fibrocartilage PCM is comprised of thin, randomly oriented collagen fibrils that entrap proteoglycans, contrasting with the densely packed, highly aligned collagen fibers in the bulk extracellular matrix (ECM). Compared to the ECM, the PCM exhibits lower modulus and greater isotropy, but has similar relative viscoelastic properties. In *Col5a1^+/−^* menisci, the reduction of collagen V results in thicker, more heterogeneous collagen fibrils, reduced modulus, loss of isotropy and faster viscoelastic relaxation in the PCM. Such altered PCM leads to impaired matrix–to–cell strain transmission, and in turn, disrupts mechanotransduction of meniscal cells, as illustrated by reduced calcium signaling activities and alters expression of matrix genes. In vitro, *Col5a1^+/−^* cells produce a weakened PCM with inferior properties and reduced protection of cells against tensile stretch. These findings highlight the PCM as a distinctive microstructure in fibrocartilage mechanobiology, underscoring a pivotal role of collagen V in PCM function. Targeting the PCM or its constituents offers potential for improving meniscus regeneration, osteoarthritis intervention and broader fibrocartilage‐related therapies.

## Graphical abstract

This study defines the structure, mechanics, and mechanobiology of the fibrocartilage pericellular matrix (PCM) using the murine meniscus, showing how collagen V deficiency alters PCM properties and disrupts cell mechanosensitive signaling. Findings emphasize the critical role of PCM in fibrocartilage mechanobiology and suggest targeting it can enhance the treatments for meniscal and other fibrocartilaginous tissue diseases.

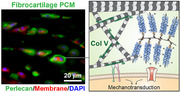

## 1 Introduction

In many biological tissues, cells inhabit a structurally distinctive microenvironment, known as the “pericellular matrix” (PCM) or “glycocalyx layer”.^[^ ^1^, ^2^, ^3^, ^4^ ^]^ Owing to its presentation directly at the cell surface, this proximal domain serves as the primary site for the initial assembly of matrix molecules, such as collagen fibrillogenesis and proteoglycan‐hyaluronan association.^[^ ^5^ ^]^ Moreover, the PCM plays a pivotal role in orchestrating cellular activities such as adhesion,^[^ ^6^ ^]^ migration,^[^ ^7^, ^8^ ^]^ growth factor sequestration,^[^ ^9^ ^]^ mechanotransduction,^[^ ^10^ ^]^ stem cell fate,^[^ ^11^ ^]^ and tumorigenesis.^[^ ^12^, ^13^ ^]^ Notably, the dense glycocalyx layer of PCM has been shown to enhance cancer cell metastasis by facilitating integrin clustering, tension and downstream integrin‐dependent signaling pathways.^[^ ^14^ ^]^ Similarly, in 3D hydrogel cultures, reciprocal interactions between human mesenchymal stem cells (hMSCs) and their PCMs drive stem cell fate and differentiation.^[^ ^15^, ^16^ ^]^ In dense connective tissues, the PCM serves as a prevalent structural feature distinct from the bulk extracellular matrix (ECM), and plays crucial roles in transmitting biomechanical cues during their extensive physiological loading. In bone, the PCM represents a ≈100 nm, perlecan‐rich pericellular space connecting the surface of bone cells, particularly osteocytes, with the bulk canalicular wall by tethering fibers.^[^ ^17^ ^]^ The PCM regulates nutrition transport, mechanosensing of fluid flow‐induced shear stress and survival of osteocytes, thereby modulating the dynamic bone adaptation to mechanical loads.^[^ ^18^, ^19^ ^]^ In articular cartilage, the PCM is ≈2–4 µm thick microdomain characterized by thinner collagen II fibrils, localization of regulatory matrix molecules such as collagen VI and perlecan, as well as lower local modulus compared to the bulk ECM.^[^ ^2^ ^]^ Cartilage PCM endows a highly negatively charged osmotic environment, essential for chondrocyte homeostasis and responses to compressive and fluid flow mechanical cues.^[^ ^2^ ^]^ In osteoarthritis (OA), the PCM was identified as the initial point of disease onset, and protecting the PCM could effectively mitigate cartilage degradation.^[^ ^20^ ^]^ In human OA, the PCM undergoes progressive loss of its molecular constituents and mechanical integrity, marked by altered concentrations of collagen VI, perlecan, and lower elastic modulus compared to normal controls.^[^ ^21^, ^22^ ^]^ In turn, the clinical significance of cartilage PCM is well appreciated,^[^ ^23^, ^24^ ^]^ as retaining the native PCM of chondrocytes has been shown to improve the quality of regenerative cartilage in OA patients.^[^ ^25^ ^]^ In contrast to bone and articular cartilage, for fibrocartilage, the structure, mechanics and mechanobiological functions of the PCM remain poorly understood.

Fibrocartilage is a family of connective tissues essential for withstanding multiaxial tensile, compressive and shear stresses in vivo. Prominent examples include the knee meniscus, temporomandibular joint (TMJ) condylar cartilage, enthesis, as well as the annulus fibrosus and endplate of the intervertebral disc (IVD).^[^ ^26^ ^]^ Reflecting its complex loading conditions, the ECM of fibrocartilage exhibits a higher degree of compositional and structural heterogeneity compared to both tension‐bearing tendon and compression‐bearing articular cartilage.^[^ ^27^, ^28^ ^]^ In fibrocartilage, the existence of the PCM and its potential role in mediating matrix–to–cell strain transmission have been acknowledged,^[^ ^29^, ^30^, ^31^ ^]^ and weakening of PCM has been noted in the annulus fibrosus of degenerative IVD.^[^ ^32^ ^]^ Despite this appreciation, the understanding of its composition, structure or mechanobiological functions remains elusive. Given the pronounced mechanosensitivity of fibrocartilage cells in homeostasis and pathogenesis,^[^ ^33^ ^]^ targeting the PCM presents a promising avenue for modulating cell mechanotransduction, improving tissue regeneration and mitigating disease progression. Such strategies hold potential for ameliorating diseases associated with fibrocartilage degeneration, such as post‐traumatic OA,^[^ ^34^ ^]^ TMJ disorder,^[^ ^35^ ^]^ disc herniation,^[^ ^36^ ^]^ and psoriatic arthritis.^[^ ^37^ ^]^

This study sought to elucidate the structure‐mechanics relationships and mechanobiological roles of fibrocartilage PCM. We studied the outer zone of murine meniscus as the model tissue. The meniscus is a loading counterpart of articular cartilage in the knee joint, whose PCM has been studied extensively,^[^ ^38^ ^]^ allowing us to highlight the distinct characteristics of fibrocartilage PCM. Leveraging the murine model enabled us to genetically perturb the PCM constituents, thereby elucidating the molecular activities governing PCM integrity. Specifically, we studied the effects of type V collagen haploinsufficiency on PCM properties and cell mechanoresponses using the *Col5a1^+/−^* murine model. Our focus on collagen V stemmed from its pivotal role as the primary nucleation site for initial fibrillogenesis of collagen I,^[^ ^39^ ^]^ the major constituent of meniscus ECM. Moreover, this collagen V‐mediated assembly predominantly occurs in the pericellular space.^[^ ^40^ ^]^ We quantified phenotypic changes in the elastic and viscoelastic micromechanics of the PCM, and modeled the impact of PCM mechanical perturbations on the matrix–to–cell strain transmission. Then, we assessed the resulting alterations in the intracellular calcium signaling activities, [Ca^2+^]*_i_*, in situ, one of the earliest and most fundamental cell responses to biophysical and biochemical stimuli.^[^ ^41^ ^]^ Next, we elucidated the impact of collagen V reduction on the gene transcriptome of meniscal cells, as well as the integrity and strain transmission function of newly assembled PCM in vitro. Together, our findings represent the first delineation of the unique molecular and functional properties of the PCM in fibrocartilage, paving the way for enhancing fibrocartilage regeneration through modulation of PCM‐mediated matrix assembly and cell mechanotransduction.

## 2 Results

### 2.1 Distinctive Composition, Nanostructure, and Micromechanics of Fibrocartilage PCM

**Figure 1:**
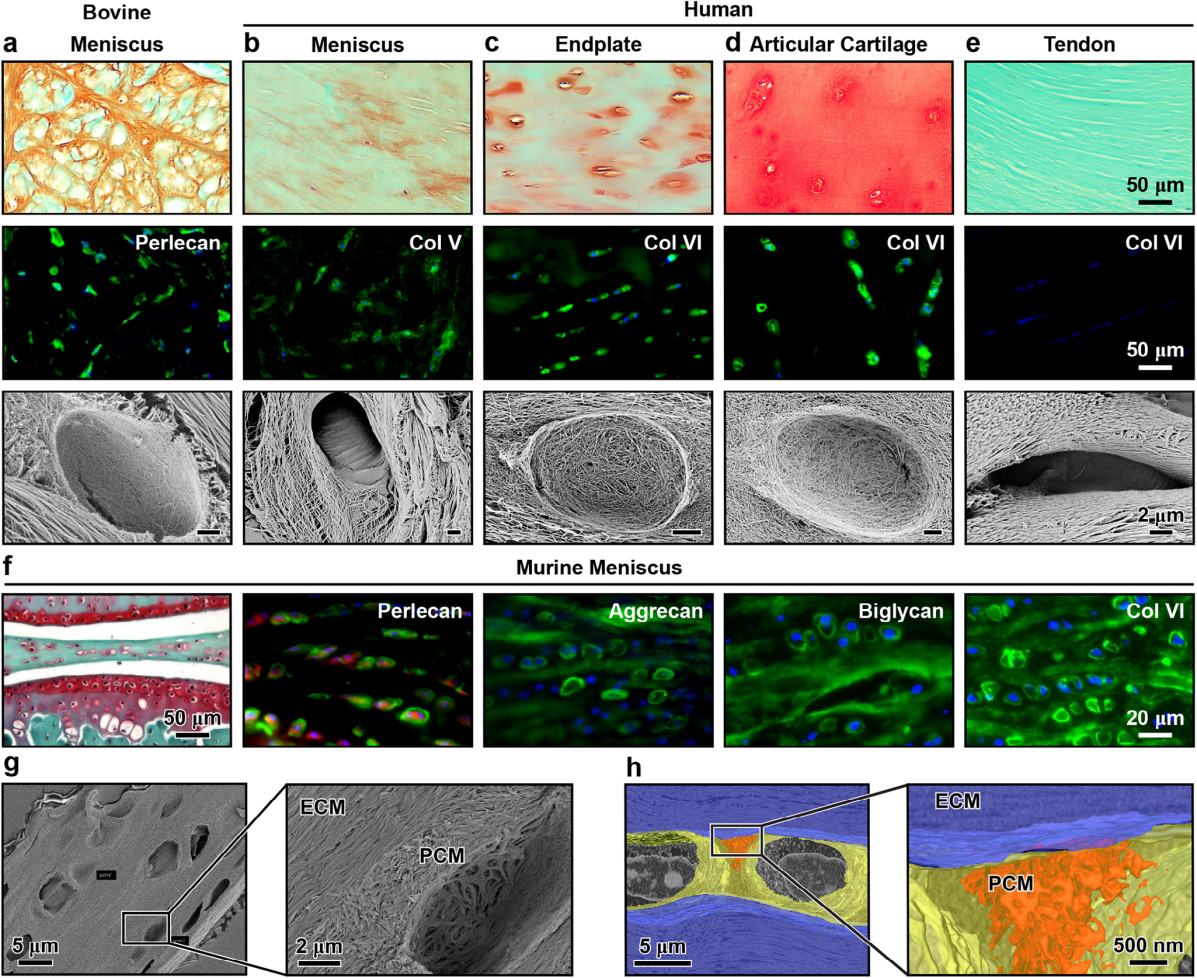
Distinct composition and nanostructure of the fibrocartilage pericellular matrix (PCM) in comparison to the bulk extracellular matrix (ECM). a–e) Molecular distribution and collagen fibril nanostructure of the PCM in fibrocartilage tissues, including a) bovine meniscus, b) human meniscus, c) human intervertebral disc (IVD) endplate, d) human articular cartilage, and e) human tendon. Note the absence of PCM domains in human tendon. First row: Distribution of sulfated glycosaminoglycans (sGAGs) via Safranin‐O/Fast Green (Saf‐O) histology. Second row: Localization and distribution of matrix molecules in the PCM, such as collagen V (Col V), collagen VI (Col VI) and perlecan via immunofluorescence (IF) imaging (blue: DAPI). Third row: Collagen fibril network nanostructure visualized by scanning electron microscopy (SEM). SEM results indicate the presence of thinner, more randomly oriented collagen fibrils in the PCM of fibrocartilage, distinct from the highly aligned, thicker collagen fiber bundles in the ECM (*n* = 3 donors, scale bar: 2 µm). f–h) Molecular composition and collagen fibril nanostructure of the meniscus fibrocartilaginous outer zone in 3‐month‐old mice. f) Localization of sGAGs via Safranin‐O histology, perlecan, aggrecan, biglycan, and collagen VI via IF imaging (red: cell membrane, blue: DAPI, *n* = 5 mice). g) Porous and randomly oriented collagen fibril network in the PCM, which is distinct from the circumferentially aligned, densely packed collagen fiber bundles in the ECM, as measured by SEM (*n* = 5), and h) by serial block‐face (SBF)‐3D SEM (orange: higher magnification showing the random fibril architecture in the PCM microdomain, yellow: PCM domain, blue: ECM domain, *n* = 3).

In various fibrocartilage tissues, the PCM exhibited distinctive composition and structure compared to the bulk ECM. As shown in bovine and human menisci, as well as human IVD endplate, the PCM was characterized by the preferred distribution of sulfated glycosaminoglycans (sGAGs) and exclusive localization of PCM biomarkers such as perlecan, collagens V and VI (**Figure** **1**a–c). Also, in these tissues, the PCM was composed of randomly oriented, thinner collagen fibrils forming a 3D basket, contrasting with the well‐aligned, densely packed collagen fibers in the bulk ECM (Figure 1a–c). In compression‐bearing articular cartilage, although the PCM differed from the bulk ECM by its higher sGAG content, thinner collagen II fibrils, and localization of collagen VI, both the PCM and bulk ECM exhibited porous, random collagen fibril networks (Figure 1d).^[^ ^42^ ^]^ This contrasted with fibrocartilage, where these two domains displayed distinct fibrillar organizations (Figure 1a–c). In tension‐bearing tendon, no distinct PCM domains were observed, and cells were found residing in spaces between the highly aligned collagen fiber bundles (Figure 1e). Therefore, the PCM of fibrocartilage was characterized by its unique composition and random, porous collagen fibril architecture, distinguishing it from the bulk fibrous ECM. Similarly, in the outer zone of murine meniscus, the PCM displayed localization of sGAGs and perlecan, as well as preferred distributions of other matrix molecules such as aggrecan, biglycan, and collagen VI (Figure 1f). At the nanoscale, collagen fibrils in the PCM also adopted a porous, randomly oriented basket‐like architecture, contrasting with the circumferentially aligned, densely packed fiber bundles in the bulk ECM (Figure 1g,h).

**Figure 2:**
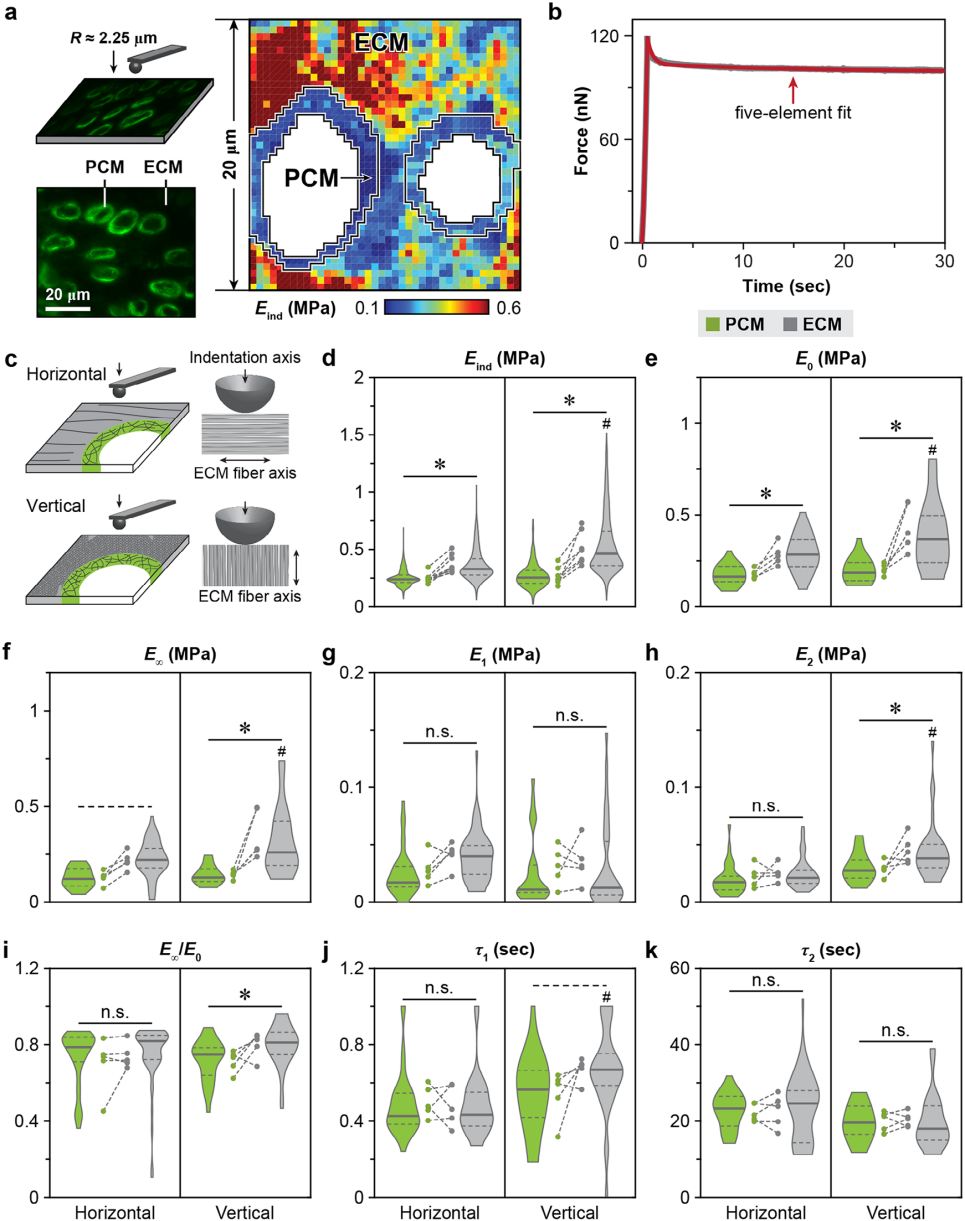
Micromechanics of the meniscus PCM and ECM via IF‐guided AFM‐nanomechanical mapping. a) Left panel: Schematic illustration of IF‐guided AFM on the horizontal cryo‐section of wild‐type (WT) murine meniscus using a microspherical tip (*R* ≈2.25 µm), where the PCM is immunolabeled with perlecan. Right panel: Representative 20 × 20 µm^2^ indentation micromodulus, *E*_ind_, map on the horizontal cryo‐section of murine meniscus. b) Representative 30 sec ramp‐and‐hold force relaxation curve, measured on the vertical cryo‐section of meniscus PCM, and corresponding five‐element model fit (R^2^ > 0.99). c) Schematic illustration of the indentation axis with respect to the fiber axis on the horizontal and vertical sections of the meniscus. d–k) Violin plots of elastic and viscoelastic micromechanical properties of 3‐month‐old WT murine meniscus PCM and ECM on horizontal and vertical cryo‐sections, including d) indentation micromodulus, *E*_ind_, e) instantaneous modulus, *E*_0_, f) equilibrium modulus, *E*_∞_, g) short‐term viscoelastic relaxation modulus, *E*_1_, h) long‐term viscoelastic relaxation modulus, *E*_2_, i) elasticity ratio, *E*_∞_/*E*_0_, j) short‐term relaxation time constant, *τ*_1_, and k) long‐term relaxation time constant, *τ*_2_. Data are pooled from ≥ 580 positions tested for *E*_ind_, ≥ 25 positions for viscoelastic properties for each group (*n* ≥ 5 mice). Each circle represents the average value from one animal. ^*^ *p* < 0.05, dashed line: *p* < 0.10, n.s.: not significant between PCM and ECM under the same indentation orientation, ^#^ *p* < 0.05 between the horizontal and vertical sections for each of the PCM or ECM domain.

Applying immunofluorescence (IF)‐guided AFM nanomechanical tests, we quantified the elastic and viscoelastic properties of the PCM in 3‐month‐old adult murine meniscus (**Figure** **2**a). We measured the effective indentation modulus, *E*_ind_, by fitting each indentation force vs depth (F‐D) curve to the Hertz model with finite thickness correction.^[^ ^43^ ^]^ Additionally, we applied the five‐element spring‐dashpot model to extract the instantaneous modulus, *E*_0_, equilibrium modulus, *E*_∞_, as well as the short‐term and long‐term relaxation moduli and time constants, (*E*_1_, *τ*_1_) and (*E*_2_, *τ*_2_) (*τ*_1_ < *τ*_2_), respectively (Figure 2b). The ratio of equilibrium‐to‐instantaneous modulus, *E*_∞_/*E*_0_, was used as an indicator of the degree of elasticity. Given the salient structural anisotropy of the fibrous ECM, we tested both horizontal and vertical cryo‐sections of the meniscus, for which, the indentation was performed perpendicular and parallel to the ECM fiber axis, respectively (Figure 2c). Our results highlighted distinct mechanical behaviors of the PCM compared to the fibrous ECM (Figure 2d–k). First, the PCM exhibited lower *E*_ind_ than the ECM on both horizontal (*E*_ind, PCM_ = 247 ± 31 kPa, *E*_ind, ECM_ = 371 ± 59 kPa, *n* = 9, mean ± 95% CI) and vertical (*E*_ind, PCM_ = 270 ± 71 kPa, *E*_ind, ECM_ = 512 ± 131 kPa, *n* = 7) sections (Figure 2d). Similar contrasts were observed for *E*_0_ and *E*_∞_ (Figure 2e,f). Second, in line with its random collagen fibril architecture and high sGAG content (Figure 1g,h), the PCM displayed isotropic mechanical properties, as we did not note significant orientation‐associated differences in either elastic or viscoelastic parameters (Figure 2d–k). This was again different from the pronounced anisotropy observed in the bulk ECM, where indentation parallel to the fiber axis on the vertical section yielded significantly higher *E*_ind_, *E*_∞_, *E*_0_, *E*_2_ and longer *τ*_1_ than that perpendicular to the fiber axis on the horizontal section (Figure 2c–k). Finally, despite the pronounced differences in moduli, the PCM exhibited only mild differences in viscoelastic behaviors from the ECM, including a lower *E*_∞_/*E*_0_ ratio (Figure 2i) and a marginally lower *τ*_1_ (Figure 2j) on the vertical section, but no other differences.

### 2.2 Collagen V Regulates the Fibril Nanostructure and Micromechanics of Meniscus PCM

**Figure 3:**
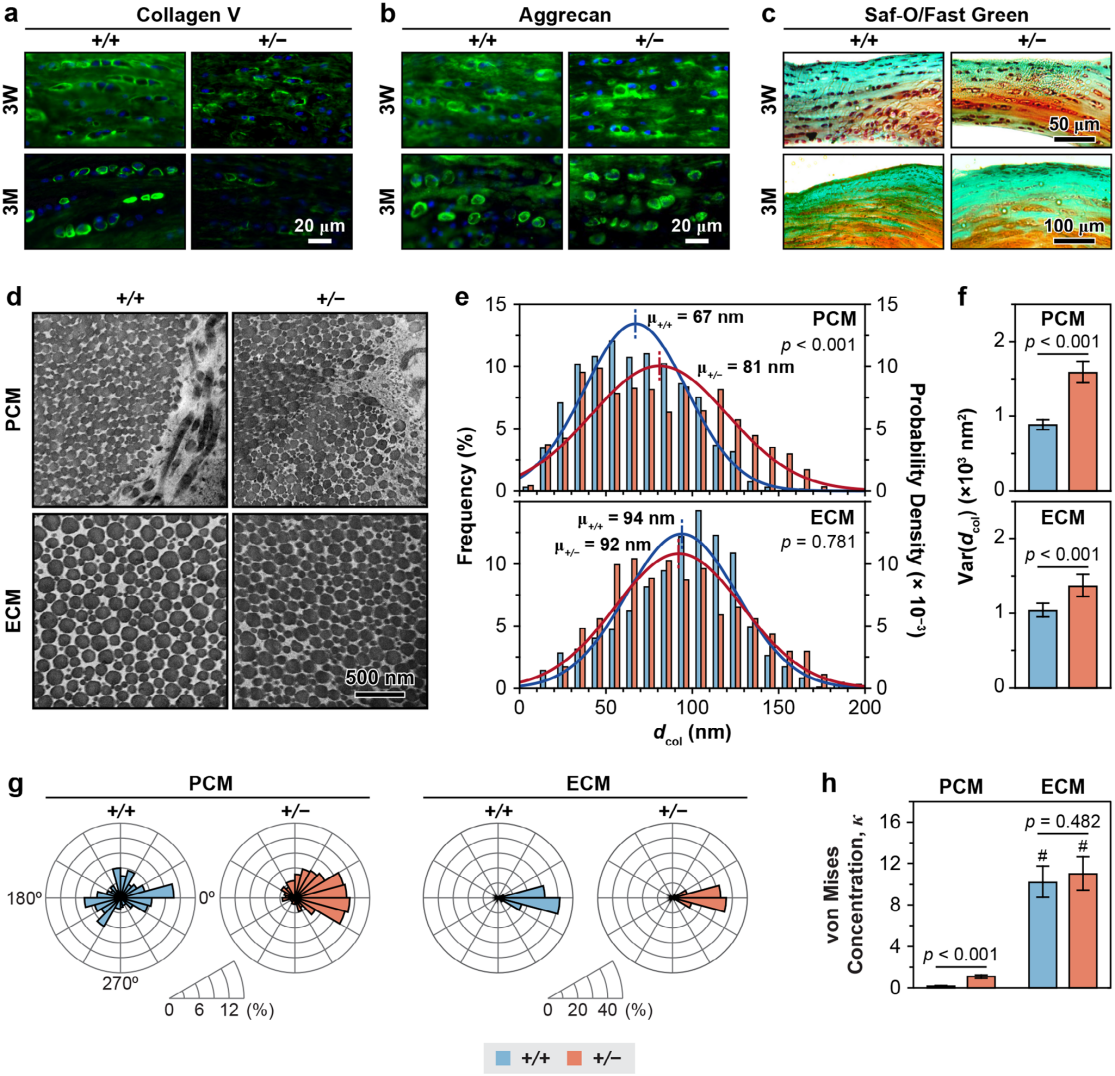
Impact of collagen V haploinsufficiency on the composition and nanostructure of the meniscus PCM. a,b) IF images show a) reduced collagen V content and b) no apparent changes of aggrecan content in the meniscus of *Col5a1^+/−^* (*+/−*) relative to that of the WT (*+/+*) control at 3‐weeks and 3‐months of ages (*n* ≥ 6 mice). c) Safranin‐O/Fast Green histology illustrates no appreciable difference in the morphology and sGAG staining between WT and *Col5a1^+/−^* murine meniscus at 3‐weeks and 3‐months of ages (*n* ≥ 6). d) Representative TEM images of collagen fibrils in the PCM and ECM of WT and *Col5a1^+/−^* meniscus, as imaged on tissue vertical sections. e) Histogram of fibril diameter distribution (≥ 640 fibrils from *n* = 5 mice for each genotype within each region). Shown together are the normal distribution, *N* (*µ*, *σ* ^2^), fits to the fibril diameter distributions (for each fit, values of *µ* and *σ* correspond to the mean and standard deviation of fibril diameters). f) Comparison of fibril heterogeneity (variance) in the PCM and ECM of WT and *Col5a1^+/−^* meniscus (mean ± 95% CI, ≥ 640 fibrils from *n* = 5 mice). g,h) Comparison of g) the fibril orientation distributions for the PCM and ECM measured on the horizontal sections of WT and *Col5a1^+/−^* meniscus, and h) the degree of fibril alignment, as denoted by the von Mises concentration parameter *κ*. Results are from > 300 fibrils pooled from *n* = 4 animals per group, with the average angle set to 0° for each sample. Results from panels d–h were measured from murine menisci at 3‐ months of age.

Then, we queried how perturbations in PCM molecular constituents impact its nanostructure and biomechanical properties by studying the phenotype of *Col5a1^+/−^* model. In both immature (3‐week‐old) and adult (3‐month‐old) *Col5a1^+/−^* murine menisci, we found preferred localization of collagen V in the PCM, alongside a noticeable reduction of collagen V compared to their age‐matched WT controls (**Figure** **3**a). Despite this reduction, there were no marked alterations in the preferred distribution of large proteoglycan, aggrecan (Figure 3b) or sGAGs (Figure 3c) within the PCM. Applying TEM and SEM to 3‐month‐old menisci, we found disrupted fibril nanostructure in the *Col5a1^+/−^* meniscus PCM, marked by substantially thickened fibrils with higher heterogeneity (variance) (Figure 3d–f). Also, in both genotypes, fibrils in the PCM were thinner than those in the bulk ECM. Meanwhile, as the fibrous ECM showed a much higher degree of fibril alignment than the PCM, as indicated by the higher von Mises concentration, *κ* (Figure 3g,h). Reduction of collagen V did not affect the degree of alignment in the ECM, but led to a significant increase in *κ* for the PCM in the *Col5a1^+/−^* meniscus. Thus, with the reduction of collagen V, the PCM developed impaired fibrillar architecture, but retained its compositional and structural distinction from the bulk ECM.

**Figure 4:**
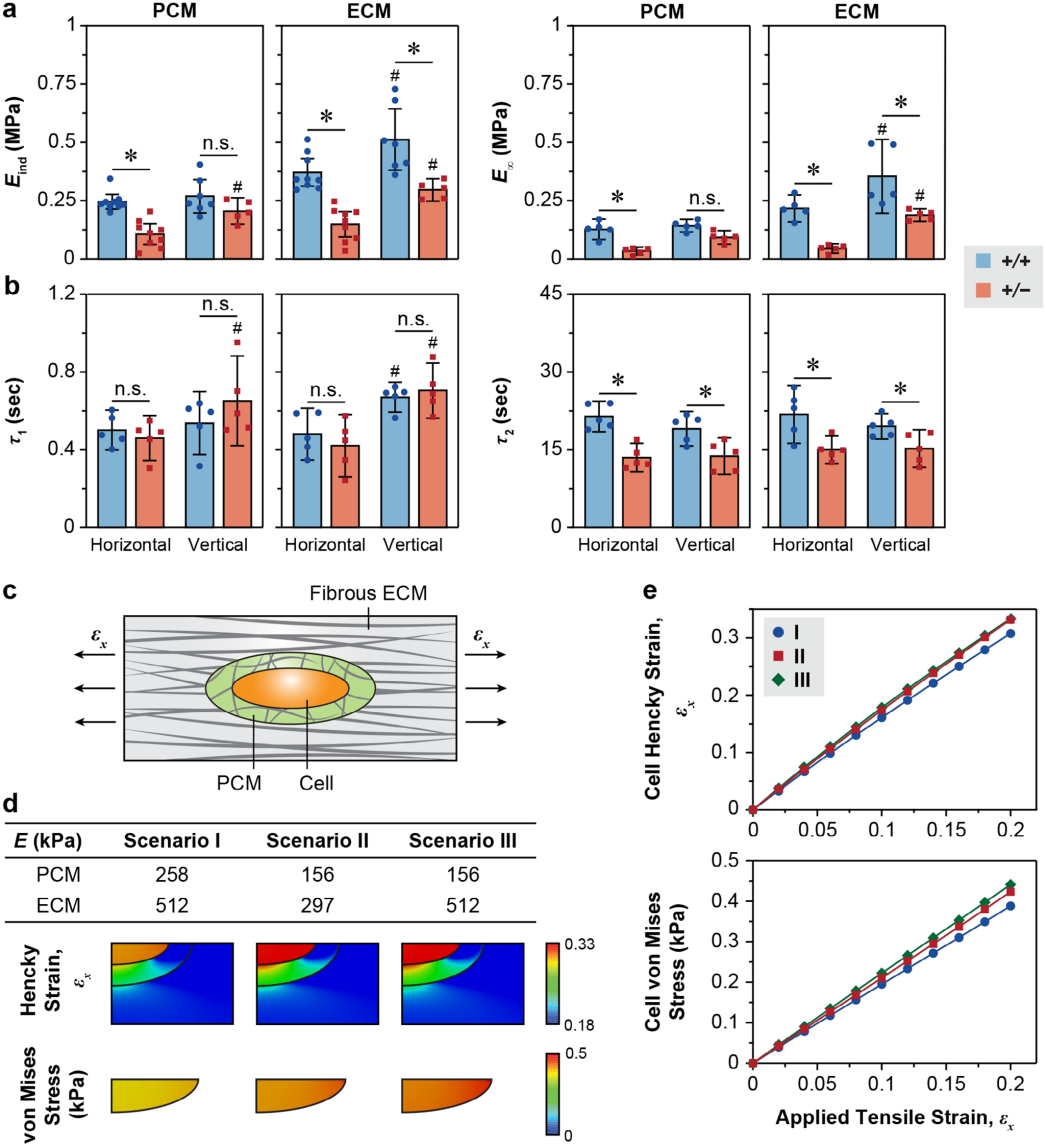
Impact of collagen V haploinsufficiency on the micromechanics and strain transduction function of the meniscus PCM. a,b) Elastic and viscoelastic micromechanical properties of the PCM and ECM on horizontal and vertical cryo‐sections, of 3‐month‐old WT (*+/+*) and *Col5a1^+/−^* (*+/−*) murine meniscus, including a) the indentation micromodulus, *E*_ind_, equilibrium modulus, *E*_∞_, and b) short term, *τ*_1_, and long term, *τ*_2_, relaxation time constants (mean ± 95% CI, *n* ≥ 5 mice). Each data point represents the average value from one animal. ^*^ *p* < 0.05 between WT and *Col5a1^+/−^* groups under the same orientation and region, ^#^ *p* < 0.05 between the horizontal and vertical sections for the same genotype and region. c–e) Finite element modeling (FEM) simulation of the PCM‐mediated strain and stress transmission to meniscal cells. c) Schematic illustration of the FEM incorporating the cell–PCM composite embedded in a fibrous ECM subjected to applied tensile stretch along the fiber axis. d) Summary of the three modeled scenarios representing normal and defective ECM and PCM mechanical properties based on the *E*_ind_ measured from WT and *Col5a1^+/−^* menisci, along with the corresponding distributions of fiber‐axis Hencky strain and cell von Mises stress under 20% applied engineering tensile strain. e) Comparison of the average cell Hencky strain and von Mises stress across the three scenarios at 0–20% applied engineering tensile strain.

In line with the disrupted fibril nanostructure, the reduction of collagen V resulted in altered PCM micromechanics, including decreased *E*_ind_, *E*_0_ and *E*_∞_ for horizontal section, as well as faster *τ*_2_ for both sections. Notably, the PCM of *Col5a1^+/−^* menisci exhibited salient anisotropy in *E*_ind_, *E*_0_, *E*_2_ and *τ*_1_ (**Figure** **4**a,b; Figure S3, Supporting Information). This result corroborated the increased fibril alignment in the PCM (Figure 3g,h), and contrasted with the isotropic micromechanical nature of the WT PCM. Meanwhile, besides its impact on the PCM, reduction of collagen V also influenced the structure and biomechanics of the bulk ECM. The ECM of *Col5a1^+/−^* menisci exhibited higher fibril diameter variance, despite having similar average diameters and fibril alignment compared to the WT ECM (Figure 3e–h). Additionally, the *Col5a1^+/−^* ECM showed decreased *E*_ind,_ *E*_0_ and *E_∞_* and faster *τ*_2_ (Figure 4a,b; Figure S3, Supporting Information), indicating a role of collagen V in overt tissue integrity beyond its impact on the PCM.

We further validated that the impaired PCM micromechanics caused by collagen V reduction led to impaired mechanical protection of the resident cells under tissue tension. We established a finite element model (FEM) that encompassed the hierarchical structure of the cell‐PCM complex embedded in a fibrous ECM bulk (Figure 4c). In this FEM, the cell and PCM were modeled as isotropic, neo‐Hookean hyperelastic materials,^[^ ^44^ ^]^ while the fibrous ECM was described by the Holzapfel‐Gasser‐Ogden (HGO) hyperelastic model,^[^ ^45^ ^]^ which is commonly used to account for tension‐compression asymmetry in fibrous biological tissues.^[^ ^46^, ^47^ ^]^ Using this model, we compared the cellular Hencky strain and von Mises stress for three scenarios under applied tensile strain along the fiber axis (Figure 4c). Scenarios I and II were simulated using the *E*_ind_ of the PCM and ECM measured from WT and *Col5a1^+/−^* menisci (Figure 4a), respectively. At 20% applied strain, the lower moduli in scenario II resulted in ≈1.08 × higher cell strain (33.2%) and ≈1.09 × higher von Mises stress (0.423 kPa) compared to those of scenario I (30.8% and 0.388 kPa) (Figure 4d,e). To delineate the direct impacts of PCM on the cellular strain and stress from those of the bulk ECM, we also tested scenario III, which adapted the defective modulus of *Col5a1^+/−^* PCM but retained the normal modulus of WT ECM. The outcomes of cellular strain and stress (33.3% and 0.441 kPa) only showed marginal differences compared to those of scenario II. Therefore, our FEM simulation supported that in *Col5a1^+/−^* meniscus, cells experienced elevated strain and stress compared to the WT meniscus under physiological‐like tensile loadings, and this effect could be primarily attributed to the impaired mechanics of the PCM, i.e., the immediate cell microniche, rather than the ECM, which is located farther away from the cells.

### 2.3 Reduction of Collagen V Disrupts PCM‐Mediated Meniscal Cell Mechanotransduction

**Figure 5:**
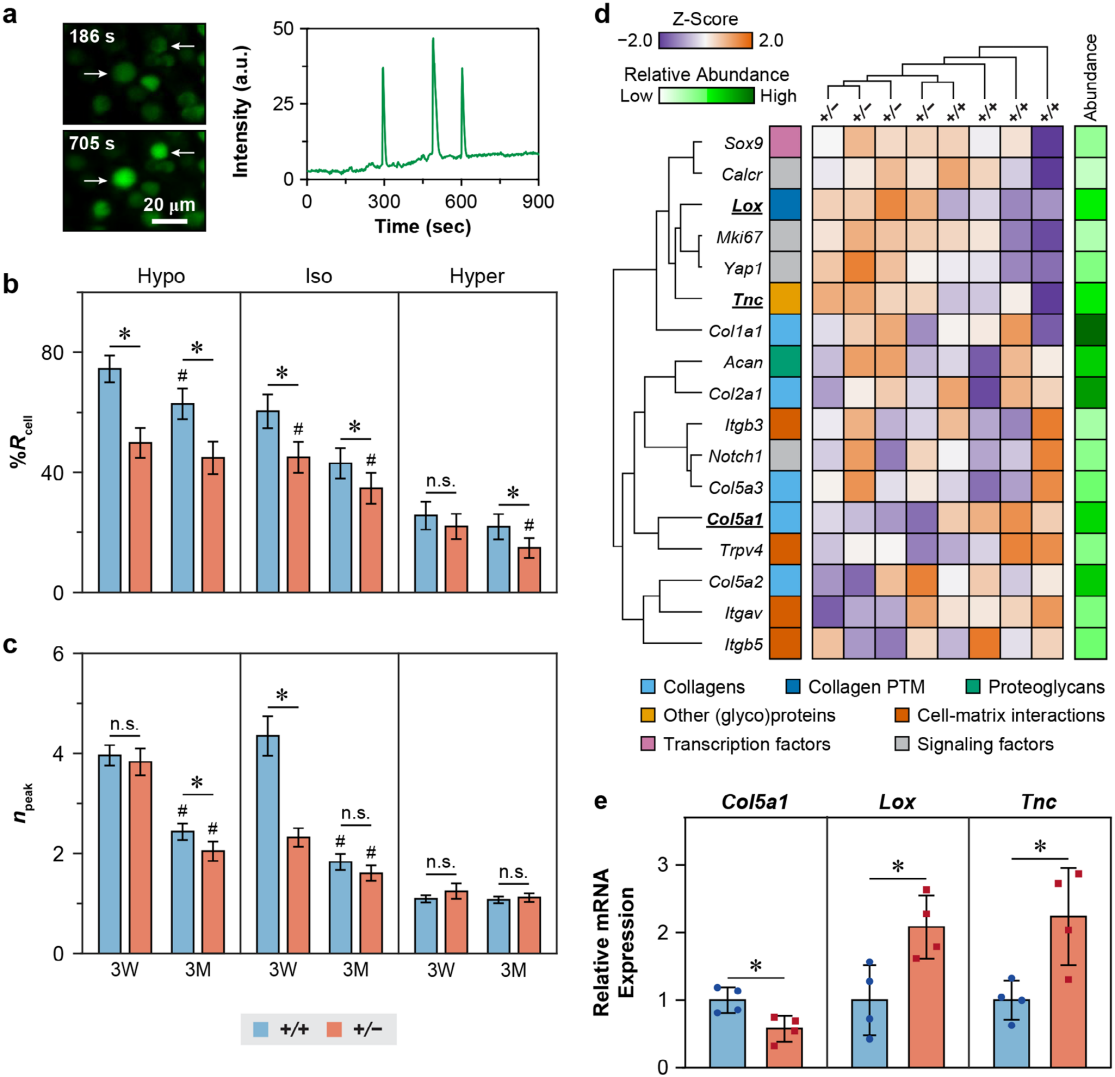
Impact of collagen V haploinsufficiency on the in situ mechanotransduction and gene expression of meniscus cells. a) Left panel: Representative confocal images of [Ca^2+^]*_i_* signaling of the WT meniscus explant in isotonic DMEM. Right panel: Representative [Ca^2+^]*_i_* oscillation fluorescence intensity curve over a 15 min time frame of a meniscal cell in hypotonic DMEM. b,c) Comparison of in situ [Ca^2+^]*_i_* signaling parameters between WT (*+/+*) and *Col5a1^+/−^* (*+/−*) at 3‐weeks and 3‐months of ages in hypotonic, isotonic and hypertonic DMEMs at 37 °C, including b) the percentage of responding cells, %*R*_cell,_ and c) number of peaks within 15 min testing time frame, *n*_peak_ (mean ± 95% CI, ≥ 65 responsive cells pooled from *n* = 4 mice for each group. ^*^ *p* < 0.05 between genotypes under same age and osmolarity, n.s.: not significant. ^#^ *p* < 0.05 between ages within same genotype and osmolarity). d) Heatmap of unbiased clustering of selected multiplexed genes for *+/+* and *+/−* menisci at 3‐weeks of age (*n* = 4 for each genotype). For the complete heatmap with a full gene list used in the cluster analysis, see Figure S4 (Supporting Information). e) Comparison of the expression of selected genes between 3‐week‐old WT and *Col5a1^+/−^* meniscus measured by quantitative PCR (mean ± S.D., *n* = 4, ^*^ *p* < 0.05). For d) and e), each biological replicate includes mRNA pooled from the eight menisci of two mice.

Next, we tested if disruption of PCM integrity due to collagen V reduction perturbs the mechanotransduction of resident meniscal cells. We studied the intracellular calcium signaling activities, [Ca^2+^]*_i_*, of freshly dissected medial meniscus explants in situ. Since the small size and irregular shape of the murine meniscus limit the application of well‐defined mechanical strains, we tested the cellular responses in both physiological (isotonic) and osmotically instigated (hypo‐ and hypertonic) Dulbecco's Modified Eagles Medium (DMEM). Using this setup, we examined both WT and *Col5a1^+/−^* menisci at 3 weeks and 3 months of age. For all tested groups, we observed spontaneous [Ca^2+^]*_i_* oscillations, from which we extracted temporal [Ca^2+^]*_i_* parameters, including the percentage of responding cells, %*R*_cell_, and the total number of [Ca^2+^]*_i_* peaks, *n*_peak_ from each responding cell over a 15 min observation period (**Figure** **5**a). Compared to the WT, *Col5a1^+/−^* cells exhibited substantially reduced [Ca^2+^]*_i_* activities, with lower %*R*_cell_ across all osmolarities and both ages, except under the hypertonic condition at 3 weeks (Figure 5b). Additionally, *Col5a1^+/−^* meniscal cells displayed lower *n*_peak_ than the WT in isotonic DMEM at 3 weeks, and hypotonic DMEM at 3 months (Figure 5c). For both genotypes, immature cells at 3 weeks showed more active [Ca^2+^]*_i_* responses than adult cells at 3 months, suggesting that collagen V deficiency did not abrogate maturation‐associated changes in cell mechanosensing. Furthermore, similar to WT cells, *Col5a1^+/−^* cells demonstrated salient osmolarity‐dependence, evidencing that loss of collagen V did not abolish cell mechanosensing in response to osmotic challenges.

Considering that meniscal cells were more mechanoresponsive at the immature age of 3 weeks (Figure 5b,c; Table S4, Supporting Information), we tested if the disrupted PCM could alter the expressions of key mechanotransduction genes by applying NanoString multiplex gene expression analysis to 3‐week‐old meniscus (*n* = 4 biological replicates/genotype), which directly counts RNA molecules within a custom‐built gene panel set. A panel set of 103 genes was tested, including major genes related to collagens, collagen post‐translational modification (PTM), proteoglycans, and other matrix proteins/glycoproteins, cell–matrix interactions, surface markers, transcriptional factors, as well as biomarkers for major mechanosensitive pathways (Table S5, Supporting Information). Besides the expected decrease in *Col5a1*, we also found significant increases in *Lox* and *Tnc* (Figure 5d; Figure S4, Supporting Information), which encode lysyl oxidase (LOX) and tenascin‐C, respectively. These gene expression changes were subsequently validated by qPCR (Figure 5e). No significant changes were detected in other tested genes. Together, these results supported that the altered PCM microenvironment resulted in perturbed mechanosensing of the resident meniscal cells, which may, in turn, alter the expressions of other matrix molecules.

### 2.4 Reduction of Collagen V Impairs the Assembly and Function of Meniscus neo‐PCM

**Figure 6:**
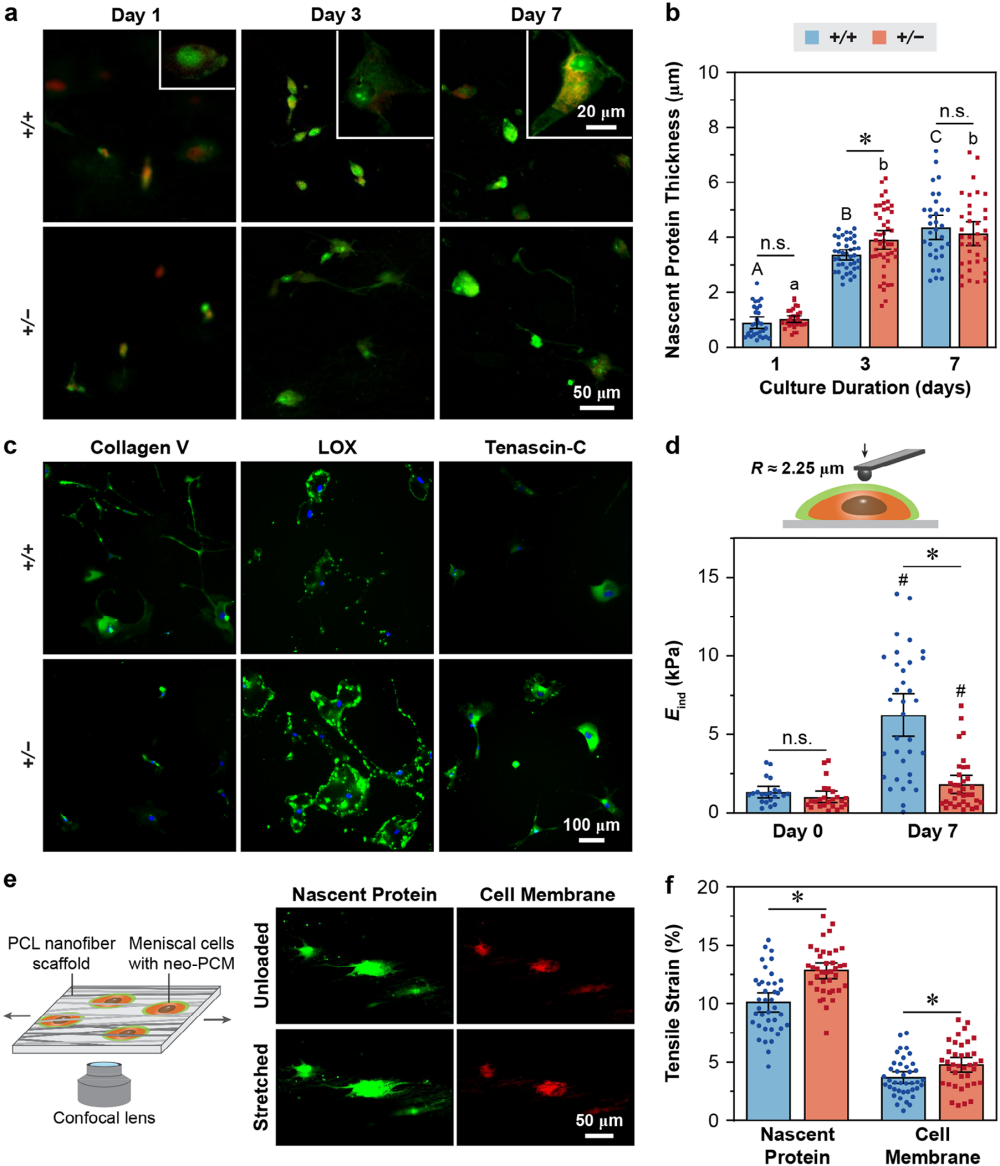
Impact of collagen V haploinsufficiency on the assembly and strain transmission function of neo‐pericellular matrix (neo‐PCM) synthesized by meniscal cells. a) Representative fluorescence images of nascent proteins (green) deposited by WT (*+/+*) and *Col5a1^+/−^* (*+/−*) meniscal cells (red: cell membrane) cultured for 7 days, as measured by metabolic click labeling of azidohomoalanine (AHA). b) Quantification of the accumulated nascent protein thickness deposited by WT and *Col5a1^+/−^* meniscus cells cultured for 1, 3 and 7 days, respectively (mean ± 95% CI, pooled from *n* ≥ 30 cells extracted from the menisci of ≥ 3 mice from three independent experiments, ^*^ *p* < 0.05 between genotypes, n.s.: not significant, different letters indicate significant differences between culture duration within each genotype). c) IF images of collagen V, lysyl oxidase (LOX) and tenascin‐C deposited by WT and *Col5a1^+−^* meniscus cells cultured for 7 days. d) Effective AFM‐nanoindentation micromodulus, *E*_ind_, of the neo‐PCM with cells shows reduced modulus for the *Col5a1^+/−^* group at 7 days, but not 0 day, of culture (mean ± 95% CI, *n* ≥ 20 cells extract from the menisci of ≥ 3 mice, ^*^ *p* < 0.05 between genotypes for each culture time point, *^#^p* < 0.05 between culture time points within each genotype, n.s.: not significant). e) Left panel: Schematic illustration of confocal imaging on the tensile stretch of meniscal cells with neo‐PCM embedded in a PCL nanofiber scaffold. Right panel: Representative confocal images of nascent protein (green) and cell membrane (red) of individual meniscal cells under 0 and 20% applied tensile stretch to the underlying PCL scaffold after culture for 7 days. f) Comparison of strain transfer from 20% applied tensile stretch to nascent protein and cell membrane between *+/+* and *+/−* meniscal cells after 7 day cultured within the PCL scaffold (mean ± 95% CI, *n* ≥ 38 cells extracted from menisci of ≥ 3 mice, ^*^ *p* < 0.05 between genotypes). Panels a–f were obtained from meniscal cells extracted from 3‐week‐old mice.

Finally, we investigated whether collagen V regulates the PCM assembly and cell–PCM interactions at the cellular level. We extracted and cultured cells from 3‐week‐old WT and *Col5a1^+/−^* menisci. Applying metabolic azidohomoalanine (AHA) “click labeling”, we assessed the distribution of newly synthesized proteins within the nascent matrix, or “neo‐PCM”, surrounding individual meniscal cells for up to 7 days in 2D culture.^[^ ^48^ ^]^ Comparing the two genotypes, we observed no significant differences in the thickness of neo‐PCM, except for a modest increase for *Col5a1^+/−^* cells at day 3 (**Figure** **6**a,b). This suggests that reduction of collagen V did not substantially alter the distribution of newly synthesized proteins during neo‐PCM formation. However, *Col5a1^+/−^* meniscal cells synthesized higher amounts of LOX and tenascin‐C proteins (Figure 6c), consistent with their increased gene expression observed in vivo (Figure 5d,e). We then applied AFM‐nanoindentation to individual cells and their neo‐PCM. At day 0, in the absence of neo‐PCM, WT, and *Col5a1^+/−^* cells showed similar apparent moduli (Figure 6d). By day 7, both genotypes showed increased apparent modulus, likely reflecting the deposition of neo‐PCM surrounding the cells. Despite similar neo‐PCM thicknesses (Figure 6b) and higher lOX levels (Figure 6c), *Col5a1^+/−^* neo‐PCM exhibited a lower micromodulus at day 7 (Figure 6d). This reduced modulus aligned with the lower modulus found in the native PCM of *Col5a1^+/−^* meniscus (Figure 4a), affirming the crucial role of collagen V in mediating the assembly and integrity of the meniscus PCM.

To test if this weakened neo‐PCM alters strain transmission, we seeded meniscal cells on the surface of an aligned porous poly(ε‐caprolactone) (PCL) nanofiber scaffold, cultured for 7 days to allow the development of neo‐PCM, and tracked its formation via AHA click labeling. The aligned PCL nanofibers simulated the circumferential fibrous architecture of the native meniscus ECM. By day 7, meniscal cells formed a composite with the neo‐PCM (Figure 6e), embedded within the > 10 µm pores of the scaffold.^[^ ^49^ ^]^ This configuration partially recapitulated the structure of native meniscus with cell–PCM units entrapped within the fibrous ECM (e.g., Figure 1), enabling us to apply tensile stretch to the residing meniscal cells and neo‐PCM. Upon applying 20% tensile strain to the PCL scaffold, we found significantly higher tensile strain along the stretch direction for *Col5a1^+/−^* neo‐PCM than that of WT (Figure 6f). Similarly, *Col5a1^+/−^* cells also showed higher strain than the WT. These results indicated that the collagen V‐deficient neo‐PCM provides impaired protection of resident cells under external tensile stretch, consistent with its reduced micromodulus (Figure 6d). This observation also corroborated our FEM simulation, which predicted higher cellular strain when cells were stretched within the native *Col5a1^+/−^* PCM compared to WT PCM (Figure 4d,e).

## 3 Discussion

**Figure 7:**
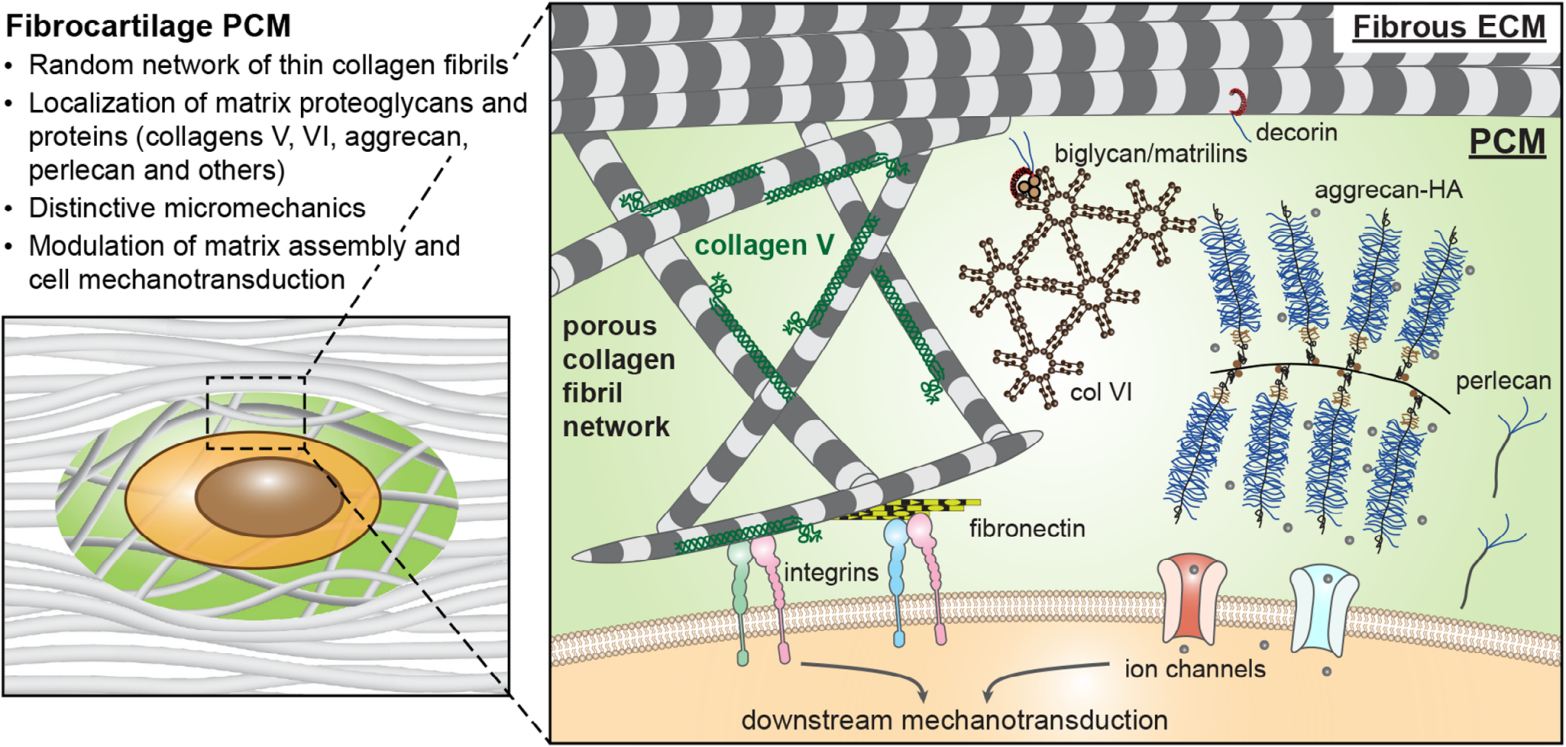
Schematic illustration of the structure and composition of fibrocartilage PCM. In fibrocartilage tissues such as the meniscus, the PCM is characterized by a random network of thinner collagen fibrils and the localization of regulatory matrix proteins and proteoglycans, resulting in isotropic micromechanical properties distinct from those of the bulk fibrous ECM. Within the PCM, the fixed negative charges endowed by proteoglycans, such as aggrecan, contribute to its micromechanical properties. In addition, collagen V is highly concentrated in the PCM, and plays pivotal roles in the fibril assembly and mechanobiological functions of the PCM.

This study underscores the PCM as a structurally distinctive microdomain across multiple fibrocartilaginous tissues (Figure 1). Comprising a porous, random fibrillar architecture in 3D with thinner collagen fibrils, preferred localization of proteoglycans and their sGAGs, the PCM stands in stark contrast to the densely packed, aligned collagen fibers of the ECM (Figure 1). Studying murine meniscus, we highlight the distinctive structure‐mechanics relationship of fibrocartilage PCM (**Figure** **7**). First, the lower modulus of the PCM relative to the ECM (Figure 2d) can be attributed to its less organized fibrillar structure, such as thinner fibrils, lower density, and higher isotropy (Figure 3d–h). While the higher sGAG content in the PCM provides additional compressive resistance through fixed charge‐endowed osmotic swelling and its confining effects of limiting collagen fibril bending or buckling,^[^ ^50^ ^]^ the impact of a less organized fibrillar structure seems to outweigh this contribution, resulting in a net lower modulus. Second, the isotropic mechanical properties of PCM stand in contrast to the salient anisotropy of the ECM (Figure 2d). For the PCM, spherical nanoindentation results in the same deformation mode on both horizontal and vertical sections, involving mutual engagement of the collagen fibril network and sGAGs.^[^ ^50^ ^]^ This isotropy arises from the random fibrillar architecture as well as the presence of sGAGs, which not only provide isotropic osmotic swelling pressure, but stretches collagen fibrils in an isotropic manner.^[^ ^51^ ^]^ In contrast, for the fibrous ECM, based on previous studies on tendon, cervix, and bovine meniscus,^[^ ^38^, ^52^ ^]^ the anisotropy originates from different deformation modes of collagen fibers under spherical nanoindentation. Indentation parallel to the fiber axis (on vertical section) leads to mutual engagement of multiple fibrils undergoing a laterally confined multiaxial compressive response, similar to a material continuum. Indentation normal to the fiber axis (on horizontal section) results in local fibril uncrimping and bending, which involve smaller shear strain transfer amongst the fibrils and are more compliant.

Despite their marked differences in structure and modulus, the PCM and ECM exhibit similar relative time‐dependent mechanical characteristics and time constants (Figure 2). The only notable difference is a mildly higher elasticity ratio *E*_∞_/*E*_0_ of the ECM on the vertical section (Figure 2i), likely due to its higher fiber packing density. In soft biological tissues, time‐dependent mechanics is governed by two mechanisms. Fluid flow‐induced poroelasticity dissipates energy through water–solid matrix interactions,^[^ ^53^ ^]^ while intrinsic viscoelasticity dissipates energy through molecular frictions during matrix macromolecular re‐configuration and is independent of fluid flow.^[^ ^54^ ^]^ Under spherical nanoindentation with an indenter tip of *R* ≈2.25 µm and a maximum indentation depth of *d*_max_ ≈100–200 nm, the characteristic fluid flow length, *L*_P_ ≈*R* × arccos[(*R* – *d*_max_)/*R*] = 0.6–1.0 µm. For the meniscus matrix, the hydraulic permeability, *k* is ≈1 × 10^−15^ *m* ^4^/(*N∙s*),^[^ ^55^ ^]^ and our nanoindentation results suggest an aggregate modulus at equillibrium, *H*_A_ ≈0.1–0.2 MPa (Figure 2f), and the poroelastic time constant *τ*_P_ is ≈*L*_P_^2^/(*H*_A_ *k*) ≈2–10 msec << 0.1 sec.^[^ ^56^ ^]^ Therefore, at this length scale, poroelasticity is expected to contribute minimally to the observed relaxation behaviors, which are dominated by intrinsic viscoelasticity. In the PCM, although thinner fibrils may expect higher molecular mobility and faster relaxation than the densely packed ECM fibers, this effect is likely offset by the confining effects of sGAGs that slow collagen fibril movement. These similarities in the relative viscoelasticity across different tissue compartments have been hypothesized to minimize local stress and strain redistribution, and reduce stress concentration at their interfaces during time‐dependent deformation.^[^ ^38^ ^]^ Such self‐consistency in time‐dependent mechanics has been observed in various heterogeneous tissue structures, including bovine meniscus across different structural units at the microscale^[^ ^38^ ^]^ and across various anatomical sites at the mesoscale,^[^ ^57^ ^]^ as well as bovine cartilage throughout tissue depth.^[^ ^58^ ^]^

The distinct structure‐mechanics relationships of the meniscus PCM represent specialized mechanical adaptation of fibrocartilage to its complex loading environment. In articular cartilage, the PCM adapts a similar structure of porous collagen II fibril networks and proteoglycans as the ECM, and its higher sGAG content compared to the ECM is central to chondrocyte mechanosensing of compressive loads^[^ ^59^, ^60^ ^]^ and protection against overloading. In tendons and ligaments, cells reside within the fibrous matrix without an intermediary PCM, except in specific cases such as within the wrap‐around tendon that also sustains compression^[^ ^61^ ^]^ and injured tendon undergoing aberrant remodeling.^[^ ^62^, ^63^ ^]^ Fibrocartilage, including the meniscus, sustains a combination of tensile and compressive loads. In the knee joint, the meniscus sustains tensile hoop stress to provide joint stability^[^ ^64^ ^]^ while transmitting femur‐tibia compressive stress to reduce cartilage loading.^[^ ^65^ ^]^ Under physiological joint loading, stretching of the circumferential ECM fibers results in multiaxial stress to the cell, including tension along the fiber axis and transverse compression perpendicular to the fiber axis.^[^ ^66^ ^]^ Therefore, sGAGs in the PCM can resist this perpendicular compression,^[^ ^67^ ^]^ and the randomly oriented collagen fibril network can redistribute tensile stress to reduce force transmitted to the cell.^[^ ^68^ ^]^ Both constituents thus contribute to attenuating cell strain and protect cells from tensile and compressive overloading (Figure 7). Indeed, our FEM simulation supports that reduced PCM micromodulus in the *Col5a1^+/−^* model contributes to increased stress and strain of resident cells under applied tension (Figure 4d,e). Similarly, the perturbed neo‐PCM assembly formed by *Col5a1^+/−^* meniscal cells in vitro leads to greater deformation of meniscal cells under tension (Figure 6e,f), underscoring impaired mechanical protection to the resident cells.

We show collagen V as a critical constituent maintaining the meniscus PCM integrity, both in native tissue (Figures 3, 4) and during in vitro cell culture (Figure 6). In *Col5a1^+/−^* meniscus PCM, the observed thickening, increased heterogeneity, and alignment of collagen fibrils (Figure 3d–h) can be attributed to the canonical role of collagen V in mediating collagen I fibrillogenesis in the pericellular space.^[^ ^39^, ^69^ ^]^ This altered fibril structure could also disrupt the integration between the fibrillar network and proteoglycans, leading to impaired strain transfer between the fibrils and reduced osmotic pressurization, ultimately resulting in lower modulus and loss of mechanical isotropy (Figure 4a,b). In addition, this disrupted fibril assembly and integration with proteoglycans contribute to higher molecular mobility for faster fibril rearrangement under stress, resulting in a shorter relaxation time, *τ*_2_ (Figure 4b). Furthermore, reduction of collagen V alters the structure and mechanics of the fibrous ECM, despite its lower concentration therein (Figure 4a,b). Such effect could also arise from the pivotal role of collagen V mediating PCM fibril assembly. During tissue development, early collagen fibrils are deposited and assembled in the pericellular space close to the cell body.^[^ ^70^ ^]^ These fibrils provide the template for growth into the mature fibrous ECM, which is located farther from the cells.^[^ ^5^ ^]^ The pre‐deposited defects due to collagen V deficiency in the pericellular fibrils thus lead to compromised ECM formation. Therefore, collagen V not only directly regulates the PCM fibril assembly, but also mediates the overall integrity of the meniscus matrix. While the *Col5a1^+/−^* mice can serve as the model for disrupted meniscus PCM, this constitutive heterozygous model does not allow us to query the impact of complete collagen V ablation due to embryo lethality of homozygous *Col5a1^−/−^* mice.^[^ ^71^ ^]^ Our future work will establish targeted collagen V knockout models by cross‐breeding *Col5a1^f/f^* mice^[^ ^72^ ^]^ with conditional inducible Cre models (e.g., *Col1a2Cre^ER^*)^[^ ^73^ ^]^ to control the timing of *Col5a1* ablation in the meniscus. This will enable us to achieve near complete ablation of collagen V without lethality and to delineate its activities in PCM versus ECM during different stages of development and remodeling. Despite this limitation, given the immediate PCM–cell contact, the observed changes in cell‐matrix mechanosensing in the *Col5a1^+/−^* meniscus (Figure 5) are expected to result from the effects of collagen V on the PCM, rather than on the ECM, which is farther removed from cells, as supported by our FEM simulation (Figure 4c–e).

We further illustrate the pivotal role of collagen V in PCM‐mediated cell mechanosensing. Specifically, the reduced in situ [Ca^2+^]*_i_* activities observed in *Col5a1^+/−^* meniscal cells (Figure 5b,c) underscore the impaired cell mechanosensing of the disrupted PCM. In contrast, multiplex gene expression analysis does not yield profoundly altered downstream signaling pathways (Figure 5d; Figure S4, Supporting Information). Cellular mechanotransduction involves a cascade of molecular events, in which, cells sense and respond to mechanical stimuli through cytoskeleton rearrangement. Cytoskeletal forces are then transmitted into the nucleus, leading to chromatin reorganization and alterations in the epigenetic landscape, thereby regulating gene expression.^[^ ^74^, ^75^ ^]^ Our results suggest that meniscal cells possess an inherently robust mechanosensing framework that preserves normal homeostasis in the presence of moderate alterations in PCM properties and transmembrane [Ca^2+^]*_i_* dynamics. This resilience may represent an adaptive biological process that enables meniscal cells to withstand extensive physiological loading. For example, following sciatic nerve resection surgery, murine meniscus was found to undergo normal postnatal growth despite substantial disruptions in joint loading and gaits.^[^ ^76^ ^]^ Also, in other tissues, collagen V plays direct biological roles such as maintaining the muscle satellite stem cell niche through the Notch‐Col V‐Calcr axis,^[^ ^77^ ^]^ influencing fibroblast growth factor‐2 (FGF‐2)‐mediated angiogenesis,^[^ ^78^ ^]^ as well as mediating cardiac and dermal wound healing by impacting integrin expression^[^ ^79^, ^80^, ^81^ ^]^ and transforming growth factor‐β (TGF‐β) signaling in fibroblasts.^[^ ^82^ ^]^ However, our multiplex gene expression analysis of the *Col5a1^+/−^* meniscus shows no substantial changes in these pathways (Figure S4, Supporting Information), suggesting either collagen V does not perform such roles in the meniscus, or that its biological activities are retained despite heterozygous reduction. Nevertheless, it is noteworthy that this impaired PCM still results in the upregulation of two crucial matrix proteins, LOX and tenascin‐C, in both native tissue (Figure 5d,e) and in vitro cell culture (Figure 6c). LOX is the key enzyme that catalyzes the conversion of collagen lysine residuals into allysine, providing the substrate for collagen cross‐link formation.^[^ ^83^ ^]^ Tenascin‐C is a glycoprotein that modulates cell–matrix interaction by attenuating the activation of focal adhesion kinase (FAK) and downstream RhoA pathway.^[^ ^84^ ^]^ These changes signify the reciprocal cellular responses to the impaired PCM microenvironment.

Our results provide new insights into fibrocartilage PCM as a promising target for improving disease intervention and tissue repair. In early OA, degradation of cartilage PCM is a well‐known early marker of disease initiation.^[^ ^20^, ^22^ ^]^ While changes of the meniscus PCM in OA have not been directly assessed, given the weakening of annulus fibrosus PCM^[^ ^32^ ^]^ and meniscus bulk ECM^[^ ^85^ ^]^ during degeneration, loss of meniscus PCM integrity could also serve as an early marker of degradation. Conversely, targeting the meniscus PCM in early OA or immediately after meniscal injury could potentially rescue its degeneration by restoring normal cell–matrix mechanosensing. In meniscus tissue engineering, mechanical stimulations are often employed to augment the biosynthesis of meniscal cells and quality of engineered products.^[^ ^33^ ^]^ Therefore, harnessing the molecular assembly and mechanobiology of the PCM could enhance the responsiveness of meniscal cells to biomechanical stimuli. Notably, in cartilage repair, the application of chondrocytes along with their native PCM, known as “chondrons”, has demonstrated positive clinical impacts in commercial applications.^[^ ^86^ ^]^ In addition, cartilage organoids have recently become an emerging tool for studying OA pathological mechanisms and therapeutics, owing to their tunable, physiological‐like 3D microenvironment and versatility in integrating with various ECM biomacromolecules, extracellular vesicles, and DNA hydrogels.^[^ ^16^, ^87^, ^88^, ^89^ ^]^ Modulating the PCM microenvironment or its key molecular constituents could serve as an effective means to harness the mechanoresponses of chondrocytes and meniscal cells in the 3D organoid culture. Given the pivotal role of collagen V in meniscus PCM highlighted by this study (Figure 7), the development of collagen V‐targeting gene therapy or biomaterials could pave a novel path toward advancing meniscus regeneration. Considering that the PCM is a prevalent feature across various fibrocartilage types, the concept of modulating the PCM or its constituents also holds promise for the regeneration and disease intervention of other fibrocartilaginous tissues, such as the annulus fibrosus,^[^ ^30^ ^]^ disc endplate (Figure 1c), and TMJ condylar cartilage.^[^ ^90^, ^91^ ^]^ In addition, while the *Col5a1^+/−^* meniscus serves as a model of defective PCM, it also represents classic Ehlers‐Danlos syndrome (cEDS), a heritable genetic disease with a prevalence of 1:20 000, caused by mutations or haploinsufficiency of the *COL5A1* or *COL5A2* gene.^[^ ^92^ ^]^ Consequently, our results provide new insights into the increased joint instability and OA propensity observed in cEDS patients,^[^ ^93^ ^]^ potentially informing improved patient care.^[^ ^94^ ^]^

## 4 Conclusion

This study identifies the pericellular matrix (PCM) as a structurally distinct microdomain that envelopes individual cells within fibrocartilage. Utilizing the murine meniscus as a model system, we demonstrate that the PCM is characterized by a random, porous collagen fibrillar network and preferred localization of proteoglycans. This structure confers distinct micromechanical properties compared to the bulk ECM, which consists of highly aligned collagen fibers, endowing the PCM with mechanoprotection of resident cells under a complex interplay of tensile and compressive loads. Studying the collagen V‐deficient model, we underscore the crucial role of collagen V in mediating the fibrillar structural integrity, mechanical properties, matrix–to–cell strain transmission and mechanobiological functions of both native PCM and neo‐PCM synthesized by meniscal cells in vitro. These findings establish collagen V as a promising target to modulate PCM assembly and cell–matrix interactions in meniscus regeneration and repair. Given the prevalence of PCM in other fibrocartilaginous tissues, the development of new regeneration or disease intervention strategies focusing on the PCM holds considerable promise for clinical translation in addressing various fibrocartilage‐associated diseases.

## 5 Experimental Section

### Human and Bovine Specimens

Human meniscus, articular cartilage, patella tendon, and intervertebral disc specimens were acquired from deidentified, healthy donors through National Disease Research Interchange (NDRI, *n* = 3 for each tissue, 47 to 71 years of age). All human sample‐related experiments were exempted for review by the determination of Drexel University Institutional Review Boards (IRB). Bovine medial menisci were harvested from knee joints of adult cows (18–30 months of age, *n* = 3, Animal Technologies). Samples were prepared for histology, immunofluorescence (IF) imaging, and collagen nanostructure analysis.

### Murine Model

Wild‐type (WT) and collagen V‐haploinsufficient (*Col5a1^+/−^*)^[^ ^39^ ^]^ mice in the C57BL/6 background were housed in the Calhoun animal facility at Drexel University. Animal use and care were approved by the Institutional Animal Care and Use Committee (IACUC) at Drexel University (Protocol #20821), following the NIH Guide for the Care and Use of Laboratory Animals. Medial menisci from 3‐month‐old *Col5a1^+/−^* and WT littermates were harvested and prepared for electron microscopy, AFM‐based nanoindentation and force relaxation tests, and intracellular calcium signaling assays. In addition, medial menisci from 3‐week‐old *Col5a1^+/−^* and WT littermates were prepared for intracellular calcium signaling assays, NanoString multiplex gene expression analysis, and in vitro cell studies. Both male and female mice were included, as no significant sex‐associated differences were detected in the histological or biomechanical properties of the meniscus.

### Histology and Immunofluorescence Imaging

Safranin‐O/Fast Green staining was applied to assess tissue morphology and sulfated glycosaminoglycan (sGAG) distribution, and IF imaging was applied to assess the presence and distribution of specific matrix molecules. Paraffin embedded human samples were sectioned via microtomy at 6 µm thickness on both transverse and sagittal planes of the meniscus, transverse plane of intervertebral disc, sagittal plane of articular cartilage, and frontal plane of patella tendon. Optimal Cutting Temperature medium (OCT) embedded bovine and murine menisci were cryo‐sectioned into 6 µm thick slices along the sagittal plane for bovine samples, and both transverse and sagittal planes for murine samples via the Kawamoto's film method.^[^ ^95^ ^]^ For IF imaging, sections were fixed in 4% paraformaldehyde (PFA) for 10 min, washed in PBS, blocked 30 min in 5% BSA, 1% goat serum buffer, followed by incubation of primary antibodies (perlecan: A7L6, 1:200 dilution, Santa Cruz Biotech; collagen V: AB7046, 1:200, Abcam; collagen VI: 70R‐CR009X, 1:200, Fitzgerald; aggrecan: AB1031, 1:200, MilliporeSigma; biglycan: LF‐159, 1:100, MilliporeSigma) at 4 °C overnight. The next day, sections were washed in PBS, incubated with secondary antibodies (perlecan: A‐11006, 1:200, Invitrogen; collagen V, collagen VI, aggrecan, biglycan: 65–6120, 1:500, Invitrogen) at room temperature for 2 h, followed by DAPI (0100‐20, SouthernBiotech) medium mounting prior to imaging (Leica DMI‐6000B). The specificity of collagen V antibody (AB7046) was validated via western blot (Figure S1, Supporting Information). Internal negative controls were prepared following the same procedure, except without primary antibody incubation (Figure S2, Supporting Information).

### Collagen Fibril Nanostructural Analysis

Scanning electron microscopy (SEM) was performed to visualize the collagen fibril nanostructure on the sections of human meniscus, articular cartilage, tendon, intervertebral disc, bovine meniscus, as well as 3‐month‐old WT murine meniscus. 6 µm thick cryo‐sections were prepared from the transverse plane of human, bovine and murine meniscus, human intervertebral disc, sagittal plane of human articular cartilage, and frontal plane of human patellar tendon. Sections were treated with 0.1% trypsin (T7409, Sigma), followed by 0.1% hyaluronidase (H3506, Sigma) at 37 °C for 24 h each, to remove proteoglycans and non‐fibrillar constituents. Samples were then fixed with Karnovsky's fixative (Electron Microscopy Sciences) at room temperature for 3 h, dehydrated in a series of graded water‐ethanol and ethanol‐hexamethyldisilazane (HMDS, A15139, Alfa Aesar) mixtures, and air dried overnight, following the established procedure.^[^ ^96^, ^97^ ^]^ SEM (Zeiss Supra 50VP) images were acquired on samples coated with ≈6 nm thick platinum. To assess the collagen fibril alignment, fibril orientation angle, *θ*, was measured from SEM images of horizontal sections via ImageJ. Values of *θ* were fitted with von Mises probability density function to calculate the von Mises concentration parameter, *κ*, a quantitative measure of fibril alignment,^[^ ^98^ ^]^ following the established procedure.^[^ ^99^, ^100^ ^]^

Serial block‐face scanning electron microscopy (SBF‐SEM) was applied to highlight the distinct 3D fibril architecture of the PCM in 3‐month‐old WT murine meniscus. Freshly dissected menisci were fixed in Karnovsky's fixative for 15 min at room temperature, and then placed on orbital shaker with gentle movement for 2 h at 4 °C. Menisci were kept in fixation solution and shipped to the University of Delaware overnight on ice without being frozen, then embedded in resin. Serial sections were performed on the transverse plane of meniscus and imaged by Apreo VolumeScope (ThermoFisher). Within each 30 × 15 × 10 µm^3^ region of interest (ROI), segmentation of the images was performed by Seg3D, then reconstituted into 3D images using Amira (ThermoFisher).

Transmission electron microscopy (TEM) was applied to quantify the collagen fibril diameter of the murine meniscus PCM and ECM. Freshly dissected menisci from 3‐month‐old WT and *Col5a1^+/−^* mice (*n* = 5/genotype) were fixed in Karnovsky's fixative for 15 min at room temperature, then placed on orbital shaker with gentle movement for 2 h at 4 °C. Menisci were kept in fixation solution and shipped to the University of South Florida overnight on ice without being frozen. Samples were rinsed with sodium cacodylate buffer and post‐fixed for 1 h with 1% osmium tetroxide, dehydrated in an ethanol series followed by 100% propylene oxide, infiltrated and embedded for 3 d in a mixture of Embed 812, nadic methyl anhydride, dodecenylsuccinic anhydride, and DMP‐30 (EM Sciences) and polymerized overnight at 60 °C.^[^ ^96^ ^]^ Ultra‐thin sections, ≈90 nm thickness, of the meniscus at frontal plane were prepared using a Leica ultramicrotome and post‐stained with 2% aqueous uranyl acetate and 1% phosphotungstic acid, pH 3.2. Sections were examined and imaged at 80 kV using a JEOL 1400 TEM (JEOL) equipped with a Gatan Orius widefield side mount CCD camera (Gatan). Based on the TEM images, collagen fibril diameter and heterogeneity were quantified via ImageJ by two independent researchers.

### IF‐Guided AFM Nanomechanical Mapping

To quantify the micromechanical properties of meniscus PCM and T/IT‐ECM, freshly dissected 3‐month‐old murine menisci were embedded in OCT medium to obtain 6 µm thick, unfixed cryo‐sections on either horizontal (transverse) or vertical (frontal) plane of the meniscus via Kawamoto's film method.^[^ ^95^ ^]^ Each cryo‐section was washed in PBS to remove excessive OCT, blocked with 10% goat serum for 30 min, and then immuno‐labeled with perlecan, the biomarker of meniscus PCM,^[^ ^29^ ^]^ by incubation with the primary perlecan antibody (A7L6, 1:50) followed by secondary antibody (A‐11006, 1:200), for 20 min each at room temperature. This immuno‐labeling procedure had been shown not to significantly alter the micromechanics of the matrix.^[^ ^101^ ^]^ On each section, two ROIs in the outer zone of the uncalcified meniscus central body were identified. The size of each ROI was 20 × 20 µm^2^, containing well‐defined PCM terrains. Guided by perlecan IF‐imaging, within each ROI, AFM‐nanomechanical mapping was executed in a 40 × 40 indentation grid within 1 × PBS at room temperature using microspherical tips (*R* ≈2.25 µm, *k* ≈0.6 N m^−1^, µMasch) up to ≈60 nN maximum indentation force at 10 µm s^−1^ indentation rate using an MFP‐3D AFM (Asylum Research), following the established procedure.^[^ ^20^, ^96^ ^]^ The effective indentation modulus, E_ind_, was calculated by fitting the entire loading portion of each indentation force–distance (F–D) curve to the finite thickness‐corrected Hertz model, assuming the Poisson's ratio *ν* ≈0.22 for the fibrous ECM,^[^ ^102^ ^]^ and ≈0.04 for the PCM.^[^ ^103^ ^]^

Within the same ROI, to quantify the viscoelastic micromechanics, ramp‐and‐hold force relaxation test was executed in a 6 × 6 grid. Following the same procedure as nanoindentation, the tip was programmed to indent the sample up to ≈60 nN maximum force at 10 µm s^−1^ *z*‐piezo displacement rate. The *z*‐piezo was then held at a constant position for ≈30 s, resulting in an approximately constant indentation depth. During this period, both the *z*‐piezo displacement and cantilever bending were recorded as a function of time at 500 Hz sampling rate, from which, the temporal profiles of indentation force, F(t), and depth, D(t) (mostly constant), were extracted. The temporal modulus, E(t) was then calculated by fitting F(t) and D(t) to the substrate corrected‐Hertz model, with the correction for taking into account the finite indentation ramp rate.^[^ ^104^, ^105^ ^]^ The relaxation of E(t) was then fitted to the 5‐element standard linear solid (SLS) model,^[^ ^38^ ^]^

(1) $$E\left(t\right)=E_{\infty }+E_{1}\exp\left(-t/\tau_{1}\right)+E_{2}\exp\left(-t/\tau_{2}\right)$$

This model yielded the equilibrium modulus, *E*_∞_, and the time‐dependent properties corresponding to the two relaxation modes, (*E*_1_, *τ*_1_) and (*E*_2_, *τ*_2_) (*τ*_1_ < *τ*_2_). The instantaneous indentation modulus, E_0_, was estimated as, E_0_ = E_∞_ + E_1_ + E_2_. The ratio of equilibrium vs instantaneous modulus, *E*_∞_/*E*_0_, as an indicator of the degree of elasticity was used. For each outcome, based on the perlecan IF‐labeling, the values corresponding to the PCM vs T/IT‐ECM were seperated using a custom MATLAB program when applicable, and values corresponding to cell remnants were excluded (e.g., cellular debris consisting of cytoplasmic organelles and nucleus damaged during sectioning).^[^ ^20^ ^]^

### Finite Element Modeling of the ECM–PCM–Cell Strain Transmission

Finite element modeling (FEM) simulations were performed using the commercial software Abaqus (Dassault Systèmes, version 2022) to elucidate the mechanical responses of the cell, PCM, and ECM under applied stretch. Due to the axisymmetric nature of the geometry and loading, all simulations were carried out in an axisymmetric modeling space to reduce computational cost. To further improve efficiency, only half of the parts were modeled, with symmetric boundary conditions applied along the axis of symmetry. The geometry was composed of three distinct regions: the cell, PCM and ECM. The cell was modeled as an ellipse positioned centrally in the domain, with a major axis of 9.5 µm and a minor axis of 5.5 µm, based on the average dimension of > 100 murine meniscal cells measured on histology images. Surrounding the cell, the PCM was represented as a concentric annular layer with 2.4 µm thickness based on the average thickness of > 100 meniscal cell PCMs. The ECM was modeled as a rectangular domain sufficiently large to minimize boundary condition effects on the FEM. A uniform stretch was applied to the outer boundary of the ECM, inducing an axial strain up to 20%, while the interfaces between all domains (cell–PCM and PCM–ECM) were defined using tie constraints to ensure perfect bonding and displacement continuity. The final model consisted of 622 CAX3 elements for the half‐cell, 960 CAX3 elements for the PCM, and 38333 CAX3 elements for the ECM, ensuring mesh convergence. All elements were 3‐node linear axisymmetric triangular elements (CAX3), allowing efficient meshing of the complex geometries while preserving numerical accuracy.

Two distinct hyperelastic material models were used to represent the constitutive behavior of the different regions. The ECM was modeled using the anisotropic hyperelastic constitutive model developed by Holzapfel, Gasser, and Ogden (HGO model) for the anisotropic mechanical behavior of fibrous ECM. This model was widely used to describe soft biological tissues with embedded collagen fiber networks exhibiting directional stiffness.^[^ ^45^ ^]^ The strain energy density function of the HGO model consisted of two components: an isotropic part representing the non‐fibrous ground matrix and an anisotropic part accounting for the aligned collagen fibers. The strain energy function *U* for the HGO model was expressed as:^[^ ^46^, ^47^ ^]^

(2) $$\begin{array}{ccc} U= & & C_{10}\left({\bar{I}}_{1}-3\right)+\frac{1}{D}\left(\frac{{\left(J^{\mathrm{el}}\right)}^{2}-1}{2}-\ln J^{\mathrm{el}}\right)\\ & & +\frac{k_{1}}{2k_{2}}\sum_{\alpha \;=\;1}^{N}\left\{\exp\left[k_{2}{\bar{E}}_{\alpha }^{2}\right]-1\right\} \end{array}$$

where C_10_ and D represented the isotropic stiffness and near‐incompressibility, respectively, and k_1_ and k_2_ were fiber‐related material parameters that defined the stiffness and nonlinearity of the collagen fibers. N was the number of families of fibers, ${\bar{I}}_{1}$ was the first invariant of $\bar{E}$ (the distortional part of the right Cauchy‐Green strain), J^el^ was the elastic volume ratio, and ${\bar{E}}_{\alpha }$ was defined as:

(3) $${\bar{E}}_{\alpha }=\kappa \left({\bar{I}}_{1}-3\right)+\left(1-3\kappa \right)\left({\bar{I}}_{4\left(\alpha \alpha \right)}-1\right)$$

where scalar parameter κ controlled the dispersion of fiber orientation. Two local directions were defined in the material orientation to represent the two default fiber families in the HGO model. The angle between the mean directions of the two fiber families was set to zero in the input file in Abaqus finite element simulations, such that both families were aligned in the same direction, effectively modeling a single dominant fiber orientation. This was consistent with experimental observations that showed the alignment of circumferential ECM fibers (Figure 1g). A rotation mapping was implemented within the input file to align the default reference direction with the direction of applied stretch, ensuring that the anisotropic reinforcement due to fibers was properly oriented relative to the mechanical loading. Model parameters were calibrated to match the values of effective indentation modulus, E_ind_, in the low‐strain regime measured on the vertical section, E = 512 kPa for the normal WT ECM, and 297 kPa for the defective (*Col5a1^+/−^*) ECM, assuming the same Poisson's ratio *ν* ≈0.22.^[^ ^102^ ^]^ The calibrated values for ECM in both normal and defective conditions were shown in **Table** **1**,

**Table 1:** Material properties of the fibrous meniscus ECM used for the Holzapfel‐Gasser‐Ogden (HGO) model.

	E (kPa)	C_10_ (kPa)	D	k_1_ (kPa)	k_2_	κ
Normal ECM	512	41.89	0.00208	51.3	3	0.15
Defective ECM	297	24.29	0.00208	51.3	3	0.15

The cell and PCM were modeled as isotropic soft materials using a neo‐Hookean hyperelastic model, which was widely employed for simulating the mechanical behavior of soft tissues and gels, particularly under moderate strains. The strain energy function U for the neo‐Hookean model was given by:^[^ ^44^ ^]^

(4) $$U=C_{10}\left({\bar{I}}_{1}-3\right)+\frac{1}{D_{1}}{\left(J^{\mathrm{el}}-1\right)}^{2}$$

where C_10_ and D_1_ were the material constant, and ${\bar{I}}_{1}$ was the first principal invariant. As shown in **Table** **2**, the model parameters C_10_ and D_1_ for the PCM were calibrated to match the average of E_ind_ experimentally measured on vertical and horizontal sections, E_ind_ = 258 kPa for the normal PCM, and 156 kPa for the defective PCM, assuming *ν* ≈0.04.^[^ ^103^ ^]^ Similarly, for the cell, the parameters were calibrated to match the average value of E_ind_ ≈1.20 kPa, as experimentally measured on WT and *Col5a1^+/−^* meniscal cells in vitro at day 0 of culture (Figure 6d), assuming *ν* ≈0.38.^[^ ^106^ ^]^

**Table 2:** Material properties of the meniscus PCM and cells used for the neo‐Hookean hyperelastic model.

	E (kPa)	C_10_ (kPa)	D_1_
Normal PCM	258	62.02	0.0214
Defective PCM	156	37.50	0.0354
Cell	1.20	0.2174	1.20

### Intracellular [Ca^2+^]*_i_* Signaling Under Osmotic Stimulation

In situ calcium signaling analysis was performed on meniscal cells from WT and *Col5a1^+/−^* mice at 3‐weeks and 3‐months of age. Freshly dissected menisci were labeled with 15 µm Calbryte 520 AM (20 650, AAT Bioquest) in DMEM (11 995 065, Gibco) with 10% (v/v) fetal bovine serum (FBS), and 1% penicillin‐streptomycin (10 378 016, Gibco) for 45 min at 37 °C, and then washed three times with DMEM prior to imaging. Intracellular calcium signaling, [Ca^2+^]*_i_*, images of cells in the outer region of the non‐ossified central body were captured every 1.5 s for 15 min in hypotonic (165 mOsm), isotonic (310 mOsm), and hypertonic (600 mOsm) DMEMs with protease inhibitors (Pierce 88 266, ThermoFisher) under a 20 × objective lens with a confocal microscope (LSM700, Zeiss). The pattern of [Ca^2+^]*_i_* oscillation was analyzed based on its fluorescent signal and transient behavior.^[^ ^107^ ^]^ In brief, the average fluorescent intensity of each cell in the images was extracted and normalized to the basal intensity value when the cell was at rest. A responsive cell was considered as any cell displaying [Ca^2+^]*_i_* signals with the peak signal exceeding four times the maximum fluctuation of the baseline. The percentage of responding cells, %*R*_cell_, was defined as the proportion of responsive cells relative to the total number of cells within a ROI. The number of [Ca^2+^]*_i_* peaks during the 15 min recording period, *n*_peak_, was extracted from all the responsive cells.

### Gene Expression Analysis via NanoString and qPCR

The expressions of key genes in the meniscal cells were assessed using the NanoString multiplex gene expression analysis. Total RNA was extracted from the central body cells of 3‐week‐old WT and *Col5a1^+/−^* menisci by homogenizing freshly dissected tissues in TRI‐reagent (T9424, Sigma) and phase‐separated in 1‐bromo‐3‐chloropropane (B9673, Sigma).^[^ ^96^ ^]^ A total of eight menisci from two siblings for each genotype were pooled as one biological replicate (*n* = 4 biological replicates/genotype) to obtain sufficient RNA. Prior to the analysis, all RNA samples were assessed to validate the purity with a 260/280 ratio between 2.0 and 2.2 by Infinite 200 PRO (Tecan) and the quality with an RNA integrity number (RIN) > 7.0 by 2100 Bioanalyzer (Agilent). NanoString multiplex gene expression analysis was performed using 100 ng of RNA per biological replicate, with custom‐designed panels targeting 103 strategically selected genes (Table S5, Supporting Information). These genes included: 1) collagens^[^ ^108^ ^]^; 2) collagens post‐translational modification (PTM)^[^ ^109^ ^]^; 3) proteoglycans^[^ ^110^ ^]^; 4) other proteins and glycoproteins^[^ ^110^ ^]^; 5) matrix remodeling^[^ ^111^ ^]^; 6) cell–matrix interactions (integrins,^[^ ^112^ ^]^ cadherins,^[^ ^113^ ^]^ transmembrane ion channels^[^ ^114^, ^115^ ^]^; and focal adhesions^[^ ^116^ ^]^); 7) cell surface markers, including adhesion molecules (*Cd44*, *Cd146*) and receptor molecules (*Cd90*, *Cd105*), 8) transcription factors. In addition, the major signaling pathways were included, such as Wnt/β‐Catenin (embryonic development, tissue homeostasis, *Bmp2*, *Bmp4*, *Dkk3*),^[^ ^117^, ^118^ ^]^ connective tissue growth factor (cell proliferation, angiogenesis, tissue fibrosis, *Ctgf*),^[^ ^119^ ^]^ RhoA (mesenchymal cell differentiation, joint tissue morphogenesis, *Gdf5*),^[^ ^120^, ^121^ ^]^ insulin‐like growth factors (mammalian growth, development, aging, *Igf1*, *Igfbp2*, *Igfbp3*),^[^ ^122^ ^]^ parathyroid related protein,^[^ ^123^ ^]^ fibroblast growth factor 3 (growth plate proliferation, chondrocyte differentiation, *Ihh*, *Pthlh*),^[^ ^123^ ^]^ transforming growth factor‐β (physiological embryogenesis, adult tissue homeostasis, *Tgfb1*, *Tgfb2*, *Tgfb3*, *Tgfbr2*),^[^ ^124^, ^125^ ^]^ and Yap/Taz (mechanotransduction, *Yap*, *Taz*),^[^ ^126^ ^]^ and cell proliferation markers (*Mki67*, *Pcna*).^[^ ^127^ ^]^ The genes of *Notch1* and *Calcr* were also included, which had been shown to be impacted by the loss of collagen V in muscle satellite stem cells.^[^ ^77^ ^]^ In addition, the gene markers for osteoblasts (*Alpl*, *Dmp1*), osteoclasts (*Tnfsf11* for RANKL, *Tnfrsf11b* for osteoprotegerin), and adipogenesis (*Pparg*) were included.^[^ ^128^, ^129^ ^]^ Low expressions for these markers were found (Figure S4, Supporting Information), which confirmed minimal contamination of cells from the ossified meniscal horns or fat pad. Outcomes were first normalized to internal positive and negative controls and subsequently normalized to the geometric mean of four housekeeping genes *Abl1*, *Actb*, *Gapdh* and *Rps17*.^[^ ^130^, ^131^ ^]^ Heatmaps were then generated using nSolver (version 4.0, Nanostring) and Matlab (version R2020a).

Quantitative RT‐PCR (qPCR) was performed to validate changes in the expressions of major genes detected by NanoString, including *Col5a1*, *Tnc* and *Lox* (primer sequences listed in Table S6 (Supporting Information)). For each biological replicate, 100 ng RNA per sample was extracted following the same procedure (*n* = 4 biological replicates/genotype), and subjected to reverse transcription using the TaqMan reverse transcription kit (N8080234, ThermoFisher), with amplification via the PowerUp SYBR Green Master Mix (A25742, ThermoFisher) on a RealPlex 4S master cycler (Eppendorf AG).

### Composition and Micromechanics of the Neo‐PCM of Meniscal Cells

Cells were extarcted from the central body of freshly dissected menisci from 3‐week‐old WT and *Col5a1^+/−^* mice by 0.2% collagenase (CLS‐2, Worthington) digestion for 5 h at 37 °C, filtered extraction solution through a 70 µm cell strainer (22 363 548, Fisher Scientific). The extracted cells were cultured overnight in cell culture flask (690 170, Greiner Bio‐One) using DMEM (11 995 065, Gibco) supplemented with 10% FBS and 1% penicillin‐streptomycin, followed by the next‐day media change to remove residual collagenase.^[^ ^48^ ^]^ For each genotype, cells extracted from a total of eight menisci from two sibling mice were pooled as one biological replicate and cultured in one flask.

For IF imaging, followed by monolayer culture (174 900, ThermoFisher) for seven days in the same DMEM with media changed every other day, cells were fixed in 4% PFA for 10 min, washed in 1 × PBS for 5 min, blocked with 5% BSA and 1% Goat Serum buffer for 30 min, followed by the incubation of primary antibodies (collagen V: AB7046, 1:200 dilution, Abcam; tenascin‐C: AB108930, 1:200, Abcam; LOX: NBP2‐24877, 1:100, Novus Biologicals) at 4 °C overnight. Samples were washed with PBS, incubated with the secondary antibody (65‐6120, 1:500, Invitrogen) at room temperature for 2 h, washed again with PBS, and then mounted with DAPI prior to imaging (Leica DMI‐6000B). Internal negative controls were prepared following the same procedure, without the incubation of primary antibodies.

To test the micromechanical properties of meniscal cells and newly synthesized PCM, 35 mm petri dish (351 008, Life Sciences) was treated with 20 µg mL^−1^ human fibronectin (10 838 039 001, Sigma‐Aldrich) for 12 h prior to seeding meniscal cells with an initial density of 5000 cells per dish. Cells were fed with DMEM (11 995 065, Gibco) with 10% FBS and 1% penicillin‐streptomycin. AFM‐nanoindentation was applied to individual meniscal cells at culture days 0 and 7 to quantify the micromechanics of meniscal cells without and with the newly synthesized matrix, respectively. For the test at day 0, cells were seeded for 3 h prior to testing to allow for cell adhesion and equilibration. The nanoindentation test was performed using microspherical tips (*k* ≈0.03 N m^−1^, *R* ≈5 µm, HQ:CSC38/tipless/Cr‐Au, cantilever B, NanoAndMore) up to ≈12 nN maximum indentation force at 10 µm s^−1^ indentation rate using a Dimension Icon AFM (Bruker Nano). The effective indentation modulus, *E*_ind_, was calculated by fitting the entire loading portion of each F‐D curve to the finite thickness‐corrected Hertz model, assuming Poisson's ratios of 0.38 for the cell^[^ ^106^ ^]^ and 0.04 for the newly synthesized cellular matrix,^[^ ^103^ ^]^ and heights of ≈5 µm for the cell^[^ ^132^ ^]^ and ≈9 µm for the cell–matrix composite (Figure 6b).

### Fabrication of Aligned Electrospun Nanofibrous Scaffolds

To test if deficiency of collagen V impaired the strain transmission of the PCM under applied tensile deformation, meniscal cells were additionally cultured on aligned poly(*ε*‐caprolactone) (PCL) nanofibrous scaffold, which mimics the highly aligned collagen fibers in meniscus ECM.^[^ ^49^, ^102^, ^133^ ^]^ First, composite fiber‐aligned fibrous scaffolds consisting of PCL and poly (ethylene oxide) (PEO) were produced by dual‐component electrospinning as previously described.^[^ ^49^ ^]^ The use of PEO here was to provide a sacrificial fiber constituent to increase the scaffold porosity (> 10 µm) and to allow the embedding of meniscal cells and their neo‐PCM within the nanofibrous scaffold during culture. In brief, PCL (80 kDa, Sigma Aldrich) was dissolved in a 1:1 mixture of tetrahydrofuran and N,N‐dimethylformamide to yield a 14.3% w/v solution (Fisher Chemical), and PEO (200 kDa, Polysciences) was dissolved in 90% ethanol to yield a 10% w/v solution. The polymer solutions were electrospun to generate 250 µm thick aligned composite fiber sheets of 50:50 PCL and PEO using a custom electrospinning device. These scaffolds were then immersed in DI water to remove the sacrificial PEO fibers, hydrated, and sterilized through a gradient of ethanol (100, 70, 50, and 30%), then coated with 20 µg mL^−1^ fibronectin for 12 h.

### Click Labeling of Nascent Proteins in the Neo‐PCM of Meniscal Cells

To image the presence and distribution of newly synthesized proteins in the neo‐matrix, following the extraction, meniscal cells were cultured at a seeding density of 5 × 10^3^ on the surface of a 1 × 1 cm^2^ PCL nanofibrous scaffold up to 7 days, fed by the “azidohomoalanine (AHA) media” with glutamine‐, methionine‐, and cystine‐free DMEM (21013‐024, Gibco), supplemented with 0.1 mm AHA (1066‐25, Click Chemistry Tools), 4 mm GlutaMAX supplement (35 050 061, Gibco), 0.2 mm L‐cystine (C7602, Sigma‐Aldrich), 100 µg mL^−1^ sodium pyruvate (11 360 070, Gibco), 50 µg mL^−1^ ascorbic acid‐2‐phosphate (A8960, Sigma‐Aldrich), 10% (v/v) FBS, and 1% penicillin‐streptomycin. To label newly synthesized matrix, at day 1, 3 and 7 of culture, cells were stained by fluorophore‐conjugated cyclooctyne (DBCO‐488, 30 µM) in 1% BSA at 37 °C for 30 min, washed with 1 × PBS wash, and fixed in 4% PFA for 30 min at room temperature, and stained for plasma membrane (CellMask Deep Red, 1:1000, Invitrogen) prior to imaging. Fluorescence images were taken using a confocal microscope (LSM700, Zeiss). ImageJ was used to quantify the nascent matrix thickness, masked by the outer edge of cell membrane, the midsection of each cell was measured radially (*n* = 5 measurements per cell) as the distance that the matrix extended, the average of 5 measurements from each cell was used to represent the nascent matrix thickness.^[^ ^48^ ^]^

### Deformation of Meniscal Cells and Neo‐PCM under Applied Tensile Strain

To test the strain transmission of the neo‐PCM under applied tensile strain, the meniscal cell‐seeded PCL scaffolds were subjected to tensile deformation using a custom tensile strain control platform integrated with the multiphoton (LSM 880, Zeiss) confocal microscope (Figure S5, Supporting Information). The platform featured a linear stage with two arms, one fixed and the other mobile. The mobile arm was driven by a one‐axis high‐load tilt platform (PT‐QX03, PDV) with a caliper micrometer head to ensure linear, gradual, and accurate movement at 20 µm increments. The meniscal cells were seeded at a density of 2 × 10^3^ on the surface of a 1 × 0.3 cm^2^ PCL scaffold, cultured for 7 days in “AHA media” to allow cell infiltration into the > 10 µm pores of the scaffold,^[^ ^49^ ^]^ followed by click labeling of newly synthesized proteins. The cell‐seeded scaffold was then mounted onto the two arms of the platform, with the central 5 mm region designated as the active loading domain. Prior to loading, a 30 µm pre‐stretch was applied to minimize slack between the scaffold and motor contacts. A 1 mm tensile stretch was then applied, resulting in ≈20% apparent tensile strain in the scaffold. The system was allowed to equilibrate for 30 sec prior to imaging. Multichannel *z*‐stack confocal images were taken on the visually tracked same ROI immediately before and after tensile stretch. During the tensile stretch and imaging, the scaffold was maintained in DMEM to preserve a near physiological environment. ImageJ was used to quantify the dimensions of the nascent protein layer and the cell membrane within the actively loaded region.

### Statistical Analysis

Linear statistical models were applied to analyze *E*_ind_ (tissue), *E*_0_, *E*_∞_, *E*_1_, *E*_2_, *τ*_1_, *τ*_2_, ratio, *d*_col_, %*R*_cell_, *n*_peak_, nascent protein thickness, and *E*_ind_ (cell), using the R package lme4 (version 1.1‐27.1).^[^ ^134^ ^]^ For continuous dependent variables, *E*_ind_ (tissue), *E*_0_, *E*_∞_, *E*_1_, *E*_2_, *τ*_1_, *τ*_2_, ratio, *d*_col_, nascent protein thickness and *E*_ind_ (cell), the linear mixed effect model (LMM) was applied. For non‐continuous variables, the generalized linear mixed model (GLMM) was applied to %*R*_cell_ (binary) with the binomial family and *n*_peak_ (count) with the Poisson family, respectively. In these tests, genotypes (*+/+* vs *+/−*), anisotropy (horizontal vs vertical), region (PCM vs ECM), age (3‐week‐old vs 3‐month‐old), osmolarity condition (hypo‐ vs iso‐ vs hypertonic) were treated as fixed effect factors when applicable, while individual animal/cell effect was treated as a randomized factor. Prior to applying linear mixed models, Shapiro‐Wilk test was applied to residuals to confirm that outcomes did not significantly deviate from normal distribution, and likelihood ratio test was applied to determine the covariance structure of the data, unstructured vs compound symmetry. For collagen structural data, *F*‐test was applied to compare fibril diameter variances, and Mardia and Jupp test of concentration equality^[^ ^98^ ^]^ was applied to compare the von Mises concentration *κ* between genotypes and between PCM and ECM, followed by the Holm‐Bonferroni correction. DESeq2 method was applied to test the gene express difference the genotypes. Unpaired two‐sample student's *t*‐test was applied to examine the differences in qPCR, strain transmission outcomes. Prior to applying student's *t*‐test, Shapiro‐Wilk test was applied to confirm that outcomes follow normal distribution. The significance level was set at *α* = 0.05. All quantitative and statistical outcomes were summarized in Tables S1–S4, S6, S7.

## Conflict of Interest

The authors declare no conflict of interest.

## Supporting information



Supporting Information

## Data Availability

The data that support the findings of this study are available from the corresponding author upon reasonable request.
