# Supplementary material for: Structure, Mechanics, and Mechanobiology of Fibrocartilage Pericellular Matrix Mediated by Type V Collagen
Source: Adv Sci (Weinh). 2025 May 23;12(32):e14750. doi: 10.1002/advs.202414750 (PMC12407281; doi:10.1002/advs.202414750)
Supplement: Supplementary file 1 — Supporting Information [file ADVS-12-e14750-s001.pdf]

ADVANCED SCIENCE
Open Access

Supporting Information

for *Adv. Sci.*, DOI 10.1002/advs.202414750

Structure, Mechanics, and Mechanobiology of Fibrocartilage Pericellular Matrix Mediated by Type V Collagen

*Chao Wang, Mingyue Fan, Su Chin Heo, Sheila M. Adams, Thomas Li, Yuchen Liu, Qing Li, Claudia Loebel, Jason A. Burdick, X. Lucas Lu, David E. Birk, Farid Alisafaei, Robert L. Mauck and Lin Han**

Supporting Information

Structure, Mechanics, and Mechanobiology of Fibrocartilage Pericellular Matrix Mediated by Type V Collagen

Chao Wang,[1] Mingyue Fan,[1] Su Chin Heo,[2] Sheila M. Adams,[3] Thomas Li,[1] Yuchen Liu,[1] Qing Li,[1] Claudia Loebel,[4] Jason A. Burdick,[5] X. Lucas Lu,[6] David E. Birk,[3] Farid Alisafaei,[7] Robert L. Mauck,[2,8] Lin Han[1,*]

[1]*School of Biomedical Engineering, Science and Health Systems, Drexel University, Philadelphia, PA 19104, United States*

[2]*McKay Orthopaedic Research Laboratory, Department of Orthopaedic Surgery, Perelman School of Medicine, University of Pennsylvania, Philadelphia, PA 19104, United States*

[3]*Department of Molecular Pharmacology and Physiology, Morsani School of Medicine, University of South Florida, Tampa, FL 33612, United States*

[4]*Department of Bioengineering, University of Pennsylvania, Philadelphia, PA 19104, United States*

[5]*BioFrontiers Institute and Department of Chemical and Biological Engineering, University of Colorado, Boulder, CO 80309, United States*

[6]*Department of Mechanical Engineering, University of Delaware, Newark, DE 19716, United States*

[7]*Department of Mechanical and Industrial Engineering, New Jersey Institute of Technology, Newark, NJ 07102, United States*

[8]*Translational Musculoskeletal Research Center, Corporal Michael J. Crescenz Veterans Administration Medical Center, Philadelphia, PA 19104, United States*

*Correspondence and requests for materials should be addressed to:

Dr. Lin Han

Phone: (215)571-3821

Fax: (215)895-4983

Email: lh535@drexel.edu.

Table S1. Summary of averaged values and statistical analysis outcomes of the micromodulus, E_{ind}, between pericellular matrix (PCM) and extracellular matrix (ECM) for 3-month-old wild-type (WT) meniscus at each region (PCM versus ECM) and anisotropy (horizontal versus vertical), shown as mean ± 95% confidence interval (CI) from values averaged by each animal.

	Horizontal					Vertical				
	PCM		ECM		p-value	PCM		ECM		p-value
	mean ± 95% CI	n	mean ± 95% CI	n		mean ± 95% CI	n	mean ± 95% CI	n	
E_{ind} (kPa)	247 ± 31	9	371 ± 59	9	0.006	270 ± 71	7	512 ± 131	7	< 0.001
E_0 (kPa)	179 ± 33	5	284 ± 71	5	0.038	204 ± 38	5	436 ± 162	5	< 0.001
E_∞ (kPa)	128 ± 44	5	217 ± 58	5	0.053	143 ± 27	5	355 ± 158	5	< 0.001
E_1 (kPa)	29 ± 17	5	41 ± 14	5	0.219	32 ± 21	5	34 ± 23	5	0.747
E_2 (kPa)	22 ± 12	5	25 ± 10	5	0.637	29 ± 8	5	46 ± 15	5	0.012
E_∞/E_0	0.70 ± 0.18	5	0.74 ± 0.08	5	0.450	0.71 ± 0.07	5	0.80 ± 0.08	5	0.209
τ_1 (sec)	0.50 ± 0.10	5	0.48 ± 0.13	5	0.751	0.54 ± 0.16	5	0.67 ± 0.08	5	0.063
τ_2 (sec)	22 ± 3	5	23 ± 5	5	0.840	20 ± 3	5	20 ± 2	5	0.671

p-value	Horizontal vs Vertical	
	PCM	ECM
E_{ind} (kPa)	0.602	0.003
E_0 (kPa)	0.595	0.004
E_∞ (kPa)	0.722	0.003
E_1 (kPa)	0.770	0.521
E_2 (kPa)	0.267	0.003
E_∞/E_0	0.801	0.397
τ_1 (sec)	0.577	0.014
τ_2 (sec)	0.251	0.389

Table S2. Summary of the distributions of collagen fibril diameter, d_{col}, and von Mises concentration, κ, and statistical analysis outcomes of the menisci from 3-month-old WT and *Col5a1*$^{+/-}$ mice, as measured by TEM and SEM, respectively.

		PCM		ECM	
		WT	*Col5a1*$^{+/-}$	WT	*Col5a1*$^{+/-}$
d_{col}	mean	67	81	94	92
	std	30	40	32	37
	Q_1	43	47	74	63
	Q_2	66	77	98	88
	Q_3	89	113	116	118
	min	8	8	11	22
	max	168	195	183	197
	$n_{fibrils}$	1,369	945	994	644
κ	mean	0.16	0.19	10.19	11.00
	95% CI	[0.10 0.22]	[0.92 1.24]	[8.75 11.75]	[9.44 12.69]
	$n_{fibrils}$	320	320	320	320

	mean(d_{col})		var(d_{col})		κ	
p-value (WT vs *Col5a1*$^{+/-}$)	PCM	ECM	PCM	ECM	PCM	ECM
	0.001	0.781	< 0.001	< 0.001	< 0.001	0.483
p-value (PCM vs ECM)	WT	*Col5a1*$^{+/-}$	WT	*Col5a1*$^{+/-}$	WT	*Col5a1*$^{+/-}$
	< 0.001	< 0.001	0.006	0.038	< 0.001	< 0.001

Table S3. Summary of averaged values and statistical analysis outcomes of the micromodulus, E_{ind}, between genotype (WT versus *Col5a1*$^{+/-}$) for 3-month-old meniscus at each anisotropy (horizontal versus vertical) and region (PCM versus ECM), shown as mean ± 95% CI from the values averaged by each animal.

	PCM									
	Horizontal					Vertical				
	WT		*Col5a1*$^{+/-}$		*p*-value	WT		*Col5a1*$^{+/-}$		*p*-value
	mean ± 95% CI	*n*	mean ± 95% CI	*n*		mean ± 95% CI	*n*	mean ± 95% CI	*n*	
E_{ind} (kPa)	247 ± 31	9	107 ± 44	9	< 0.001	270 ± 71	7	205 ± 57	5	0.149
E_0 (kPa)	179 ± 33	5	57 ± 25	5	0.002	204 ± 38	5	136 ± 28	5	0.062
E_∞ (kPa)	128 ± 44	5	35 ± 17	5	0.008	143 ± 27	5	93 ± 29	5	0.140
E_1 (kPa)	29 ± 17	5	12 ± 9	5	0.080	32 ± 21	5	15 ± 13	5	0.128
E_2 (kPa)	22 ± 12	5	10 ± 7	5	0.074	29 ± 8	5	25 ± 11	5	0.521
E_∞/E_0	0.70 ± 0.18	5	0.64 ± 0.12	5	0.248	0.71 ± 0.07	5	0.70 ± 0.10	5	0.813
τ_1 (sec)	0.50 ± 0.10	5	0.46 ± 0.12	5	0.594	0.54 ± 0.16	5	0.65 ± 0.23	5	0.134
τ_2 (sec)	22 ± 3	5	15 ± 3	5	< 0.001	20 ± 3	5	15 ± 3	5	0.007

	ECM									
	Horizontal					Vertical				
	WT		*Col5a1*$^{+/-}$		*p*-value	WT		*Col5a1*$^{+/-}$		*p*-value
	mean ± 95% CI	*n*	mean ± 95% CI	*n*		mean ± 95% CI	*n*	mean ± 95% CI	*n*	
E_{ind} (kPa)	371 ± 59	9	149 ± 54	9	< 0.001	512 ± 131	7	297 ± 48	5	< 0.001
E_0 (kPa)	284 ± 71	5	73 ± 38	5	< 0.001	436 ± 162	5	260 ± 44	5	< 0.001
E_∞ (kPa)	217 ± 58	5	46 ± 21	5	< 0.001	355 ± 158	5	188 ± 27	5	< 0.001
E_1 (kPa)	41 ± 14	5	16 ± 6	5	0.007	34 ± 23	5	29 ± 29	5	0.550
E_2 (kPa)	25 ± 10	5	13 ± 10	5	0.078	46 ± 15	5	42 ± 24	5	0.554
E_∞/E_0	0.74 ± 0.08	5	0.66 ± 0.11	5	0.109	0.80 ± 0.08	5	0.73 ± 0.12	5	0.192
τ_1 (sec)	0.48 ± 0.13	5	0.42 ± 0.16	5	0.436	0.67 ± 0.08	5	0.71 ± 0.14	5	0.654
τ_2 (sec)	23 ± 5	5	16 ± 3	5	0.001	20 ± 2	5	16 ± 4	5	0.024

p-value	Anisotropy (Horizontal vs Vertical)				Region (PCM vs ECM)			
	WT		*Col5a1*$^{+/-}$		WT		*Col5a1*$^{+/-}$	
	PCM	ECM	PCM	ECM	Horizontal	Vertical	Horizontal	Vertical
E_{ind} (kPa)	0.543	0.001	0.026	0.001	< 0.001	< 0.001	0.118	0.016
E_0 (kPa)	0.481	< 0.001	0.034	< 0.001	0.007	< 0.001	0.643	0.002
E_∞ (kPa)	0.638	< 0.001	0.085	< 0.001	0.010	< 0.001	0.734	0.007
E_1 (kPa)	0.752	0.487	0.578	0.115	0.107	0.667	0.757	0.121
E_2 (kPa)	0.319	0.005	0.034	< 0.001	0.604	0.007	0.580	0.006
E_∞/E_0	0.779	0.270	0.231	0.160	0.188	0.014	0.552	0.352
τ_1 (sec)	0.636	0.016	0.016	0.001	0.783	0.083	0.597	0.477
τ_2 (sec)	0.201	0.208	0.871	0.905	0.686	0.66	0.143	0.163

Table S4. Summary of averaged values and statistical analysis outcomes of $[Ca^{2+}]_i$ signaling parameters between genotype (WT versus *Col5a1*$^{+/-}$) for meniscal cells at each osmolarity (hypotonic, isotonic and hypertonic) and age (3 weeks versus 3 months), shown as mean ± 95% CI from the values averaged by cells.

	$\%R_{cell}$					
	3 weeks			3 months		
mean ± 95% CI	Hypotonic	Isotonic	Hypertonic	Hypotonic	Isotonic	Hypertonic
WT	74 ± 5	60 ± 6	26 ± 5	63 ± 5	43 ± 5	22 ± 4
Col5a1$^{+/-}$	50 ± 5	45 ± 5	22 ± 4	45 ± 5	35 ± 5	15 ± 3
p-value (genotype)	< 0.001	< 0.001	0.265	< 0.001	0.024	0.009
p-value (osmolarity)	Hypo vs Iso	Hypo vs Hyper	Iso vs Hyper	Hypo vs Iso	Hypo vs Hyper	Iso vs Hyper
WT	< 0.001	< 0.001	< 0.001	< 0.001	< 0.001	< 0.001
Col5a1$^{+/-}$	0.391	< 0.001	< 0.001	0.023	< 0.001	< 0.001

	n_{peak}					
	3 weeks			3 months		
mean ± 95% CI	Hypotonic	Isotonic	Hypertonic	Hypotonic	Isotonic	Hypertonic
WT	3.96 ± 0.20	4.35 ± 0.39	1.09 ± 0.07	2.44 ± 0.17	1.83 ± 0.16	1.07 ± 0.07
Col5a1$^{+/-}$	3.83 ± 0.26	2.32 ± 0.19	1.25 ± 0.16	2.05 ± 0.19	1.61 ± 0.16	1.12 ± 0.09
p-value (genotype)	0.487	< 0.001	0.358	0.017	0.166	0.788
p-value (osmolarity)	Hypo vs Iso	Hypo vs Hyper	Iso vs Hyper	Hypo vs Iso	Hypo vs Hyper	Iso vs Hyper
WT	0.122	< 0.001	< 0.001	< 0.001	< 0.001	< 0.001
Col5a1$^{+/-}$	< 0.001	< 0.001	< 0.001	0.028	< 0.001	0.023

p-value (age)	$\%R_{cell}$			n_{peak}		
	Hypo	Iso	Hyper	Hypo	Iso	Hyper
WT	0.001	< 0.001	0.251	< 0.001	< 0.001	0.902
Col5a1$^{+/-}$	0.188	0.006	0.008	< 0.001	< 0.001	0.479

Table S5. List of genes for multiplex gene expression analysis via NanoString.

Collagens	Collagen PTM	Proteoglycans	Other proteins/ glycoproteins	Matrix remodeling
Col1a1, a2	*Plod1,2a,3*	*Acan*	*Acta2*	*Adamts1,4,5*
Col2a1	*Lox*	*Bgn*	*Comp*	*Mmp2,3,9,13*
Col3a1	*Loxl2*	*Dcn*	*Fn1*	*Timp1,3*
Col5a1,a2,a3	*Adamts2*	*Fmod*	*Halpn1*	
Col6a1,a2,a3	*Bmp1*	*Hspg2*	*Has2*	
Col9a1,a2,a3		*Lum*	*Matn1,3*	
Col10a1		*Vcan*	*Prg4*	
Col11a1,a2			*Tnc*	

Cell-matrix interactions	Cell surface markers	Transcription factors	Signaling factors	Osteoblast/ osteoclast/ adipogenesis biomarkers
Cdh2,3,5,11	*Cd44*	*Mkx*	*Bmp2,4*	*Alpl*
Itga1,3,5,v	*Cd90*	*Rbpj*	*Calcr*	*Dmp1*
Itgb1,3,5	*Cd105*	*Runx2*	*Ctgf*	*Tnfsf11*
Piezo1,2	*Cd146*	*Scx*	*Dkk3*	*Tnfrsf11b*
Trpv4		*Sox9*	*Gdf5*	*Pparg*
Postn			*Igf1*	
Pxn			*Igfbp2,3*	
Vcl			*Ihh*	
			Notch1	
			Pthlh	
			Tgfb1,2,3,r2	
			Yap1, Taz	
			Mki67, Pcna	

Housekeeping genes: *Abl1, Actb, Gapdh, Rps17*

Table S6. List of primers for qPCR and a summary of averaged values and statistical analysis outcomes of qPCR between genotype (WT versus *Col5a1*$^{+/-}$) for 3-week-old meniscus.

Gene	Forward Primer	Reverse Primer
Col5a1	5'-AAGCGTGGGAAACTGCTCTCCTAT-3'	5'-AGCAGTTGTAGGTGACGTTCTGGT-3'
Lox	5'-ACGGCTACCACAGAAGCTG-3'	5'-ATGGCTGTTGTTGCTATGGCA-3'
Tnc	5'-TCTTCTGCTGCGTGACAACC-3'	5'-GAGAAACCAGCTTGGAACCAG-3'
β-actin	5'-AGATGACCCAGATCATGTTTGAGA-3'	5'-CACAGCCTGGATGGCTACGT-3'

	WT		*Col5a1*$^{+/-}$		*p*-value (genotype)
	mean ± 95% CI	*n*	mean ± 95% CI	*n*	
Col5a1	1.00 ± 0.30	4	0.58 ± 0.30	4	0.020
Lox	1.00 ± 0.82	4	2.08 ± 0.74	4	0.021
Tnc	1.00 ± 0.46	4	2.24 ± 1.14	4	0.034

Table S7. Summary and statistical analysis outcomes of *in vitro* analysis between genotypes (WT versus *Col5a1*$^{+/-}$) for 3-week-old meniscus.

		WT		*Col5a1*$^{+/-}$		p-value (genotype)
		mean ± 95% CI	n_{cell}	mean ± 95% CI	n_{cell}	
Nascent protein thickness (μm)	day 1	0.90 ± 0.21	31	1.02 ± 0.12	32	0.644
	day 3	3.37 ± 0.18	40	3.91 ± 0.34	46	0.011
	day 7	4.37 ± 0.44	32	4.14 ± 0.43	36	0.381
neo-PCM modulus (kPa)	day 0	1.34 ± 0.36	23	1.03 ± 0.37	24	0.663
	day 7	6.25 ± 1.36	34	1.83 ± 0.57	35	< 0.001
Tensile strain	Nascent protein	10.10 ± 0.82	40	12.83 ± 0.68	38	< 0.001
	Cell membrane	3.67 ± 0.51	40	4.77 ± 0.63	38	0.008

p-value (time points)	Nascent protein thickness			neo-PCM modulus
	day 1 vs 3	day 1 vs 7	day 3 vs 7	day 0 vs 7
WT	< 0.001	< 0.001	< 0.001	< 0.001
Col5a1$^{+/-}$	< 0.001	< 0.001	0.629	0.209

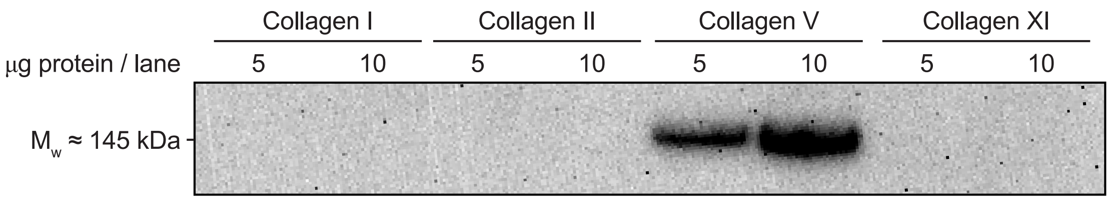

Figure S1. Western blot on recombinant murine collagen I, collagen II extracted from human cartilage, collagen V extracted from bovine skin and collagen XI extracted from fetal bovine cartilage, validated the specificity of collagen V antibody (AB7046).

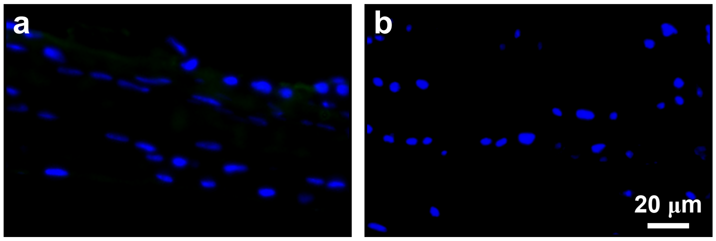

Figure S2. Internal negative control for the immunofluorescence imaging analysis. The controls were incubated with secondary antibodies but no primary antibody incubation. Panel a: secondary antibody goat anti-rabbit, b: secondary antibody goat anti-rat, blue: DAPI.

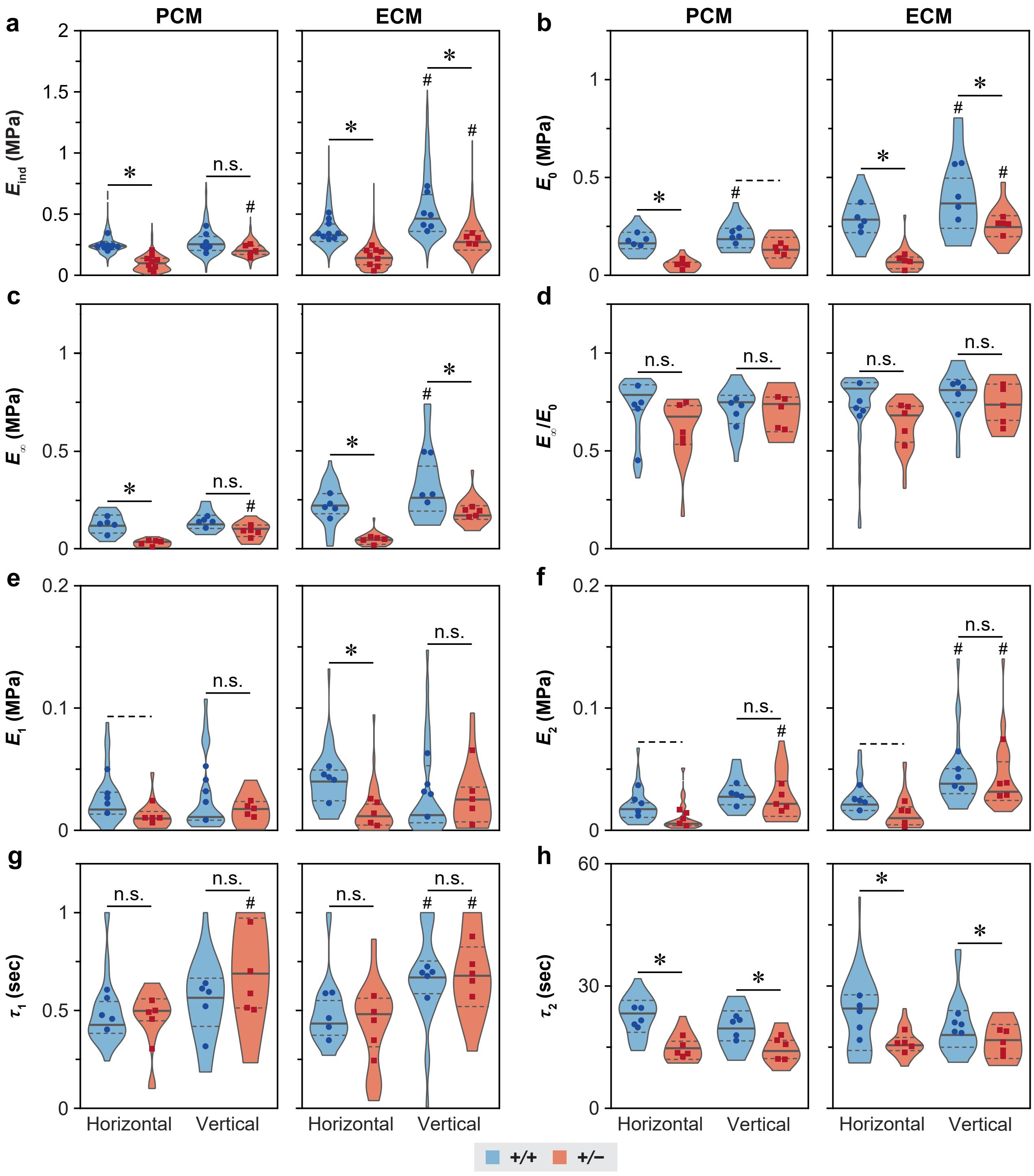

Figure S3. Violin plots of elastic and viscoelastic micromechanical properties of meniscus PCM and ECM from 3-month-old WT (+/+) and *Col5a1*$^{+/-}$ (+/−) mice on horizontal and vertical cryo-sections, a) indentation micromodulus, E_{ind}, b) instantaneous modulus, E_0, c) equilibrium modulus, E_∞, d) elasticity ratio, E_∞/E_0, e) short-term viscoelastic relaxation modulus, E_1, f) long-term viscoelastic relaxation modulus, E_2, g) short-term relaxation time constant, τ_1, and h) long-term relaxation time constant, τ_2. Data are pooled from ≥ 580 positions tested for E_{ind}, ≥ 25 positions for viscoelastic properties for each group ($n \geq 5$ mice). Each data point represents the average value from one animal. *: $p < 0.05$, dashed line: $p < 0.10$, n.s.: not significant between WT and *Col5a1*$^{+/-}$ groups for the same orientation and region, $^{\#}$: $p < 0.05$ between the horizontal and vertical sections for the same genotype and region. Results were measured from murine menisci at 3 months of age.

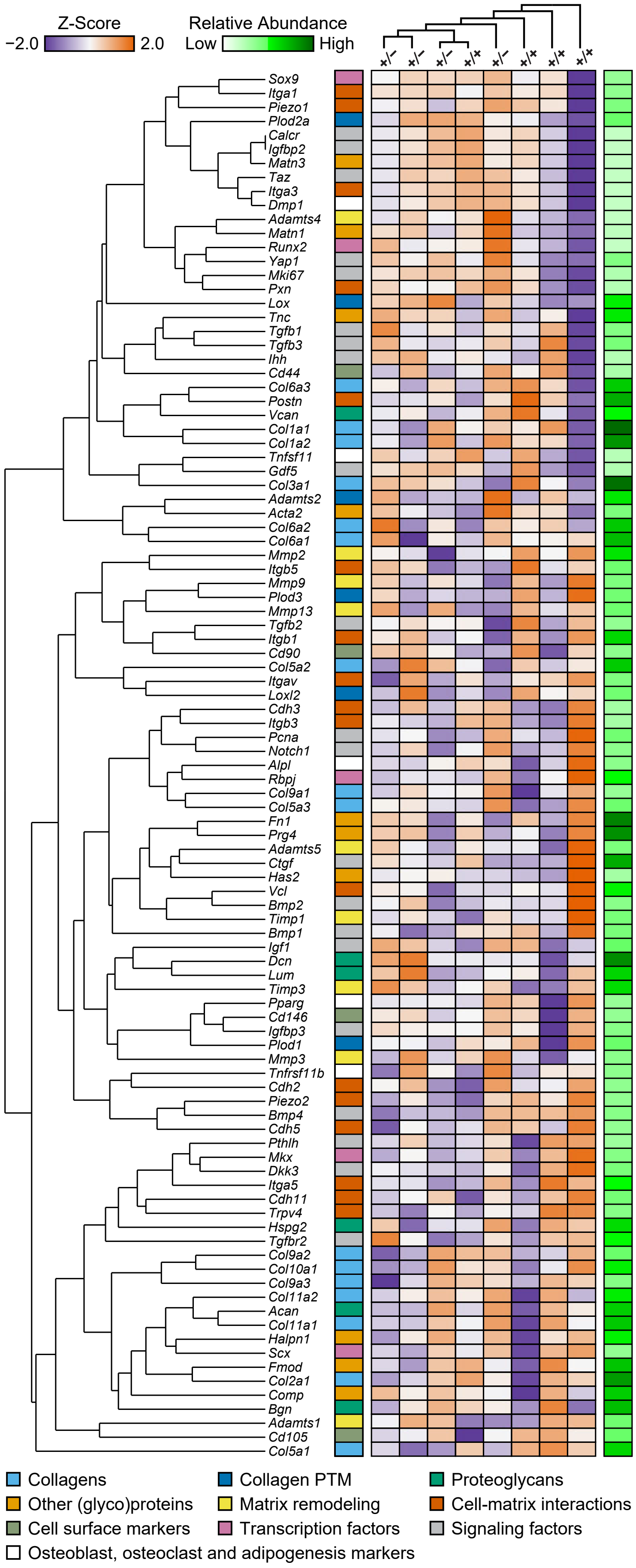

Figure S4. Heatmap showing hierarchical clustering of the full multiplexed gene set for WT (+/+) and *Col5a1*$^{+/-}$ (+/−) murine menisci at 3 weeks of age (n = 4 for each genotype).

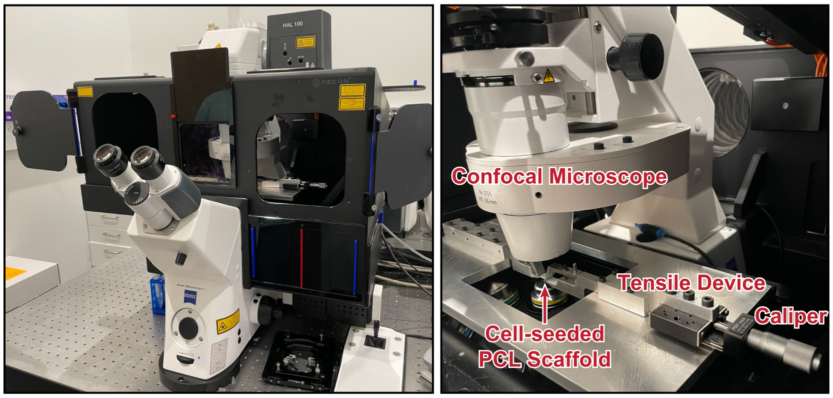

Figure S5. Experimental setup of the customized tensile device under the multiphoton confocal imaging system. Cell-seeded PCL was immersed in DMEM, and tensile test was controlled by a caliper of the tensile device. The same regions of interests (ROIs) on the PCL before and after 20% tensile strain were imaged by the multiphoton confocal microscope (LSM 880, Zeiss).
